# Synthesis and Fluorescent Properties of Novel Isoquinoline Derivatives

**DOI:** 10.3390/molecules24224070

**Published:** 2019-11-10

**Authors:** Łukasz Balewski, Franciszek Sączewski, Maria Gdaniec, Anita Kornicka, Karolina Cicha, Aleksandra Jalińska

**Affiliations:** 1 Department of Chemical Technology of Drugs, Faculty of Pharmacy, Medical University of Gdańsk, 80-416 Gdańsk, Poland; saczew@gumed.edu.pl (F.S.); anita.kornicka@gumed.edu.pl (A.K.); cicha.karolina89@gmail.com (K.C.); aleksandra.jalinska@gumed.edu.pl (A.J.); 2 Faculty of Chemistry, Adam Mickiewicz University, 61-614 Poznań, Poland; magdan@amu.edu.pl

**Keywords:** isoquinoline derivatives, copper-catalyzed coupling, fluorescence, X-ray crystallography

## Abstract

Isoquinoline derivatives have attracted great interest for their wide biological and fluorescent properties. In the current study, we focused on the synthesis of a series of novel isoquinoline derivatives substituted at position 3 of the heteroaromatic ring. Compounds were obtained in a Goldberg–Ullmann-type coupling reaction with appropriate amides in the presence of copper(I) iodide, *N*,*N*-dimethylethylenediamine (DMEDA), and potassium carbonate. The structures of novel isoquinolines were confirmed by IR, NMR, and elemental analysis, as well as X-ray crystallography. In the course of our research work, the visible fluorescence of this class of compounds was observed. The above findings prompted us to investigate the optical properties of the selected compounds.

## 1. Introduction

Among heteroaromatic compounds, the isoquinoline (benzo[*c*]pyridine) scaffold constitutes an important and privileged structural framework, and may be found in several naturally occurring alkaloids [1,2]. Biological activities of isoquinoline derivatives found in nature, as well as those obtained by organic synthetic procedures, have been widely explored. Isoquinoline compounds possess antihypertensive, anti-inflammatory, anti-oxidant, antipyretic, and analgesic properties [3,4,5]. It was also demonstrated that isoquinoline derivatives exhibit antifungal, antibacterial [6], and anti-malarial activities [7]. In addition, it was found that isoquinolines may act as antidepressants and antipsychotic agents [8]. Several compounds with isoquinoline core were found to exhibit anti-tumor or antiproliferative activity serving as a lead structure for the development of potential anticancer drugs [9,10,11,12]. Noteworthy is the fact that the isoquinoline ring constitutes an important molecular part of topical anesthetic drug quinisocaine, whereas the 1,2,3,4-tetrahydroisoquinoline moiety is found in the structure of antihypertensive drugs, quinapril and debrisoquine.

An inspection of the literature data indicates that isoquinoline-3-amine derivatives exhibit fluorescent properties and may constitute potential fluorophores [13,14,15]. Moreover, for many years the electronic states and optical properties of isoquinoline and its derivatives have been an object of particular interest and study [16,17,18,19,20,21,22,23,24,25]. Recently, notable progress has been made in the synthesis and development of novel organic fluorophores with diverse applications and uses in in vivo imaging. Derivatives with high extinction coefficients, long absorption wavelengths, high quantum yields of fluorescence, and high fluorescence lifetimes may be obtained by molecular design [26].

## 2. Results and Discussion

### 2.1. Synthesis of 1-(Isoquinolin-3-yl)heteroalkyl(aryl)-2-ones ***3a***-***d*** and 1,3-di(Isoquinolin-3-yl)heteroalkyl(aryl)-2-ones ***4a***-***c***

**Scheme 1:**
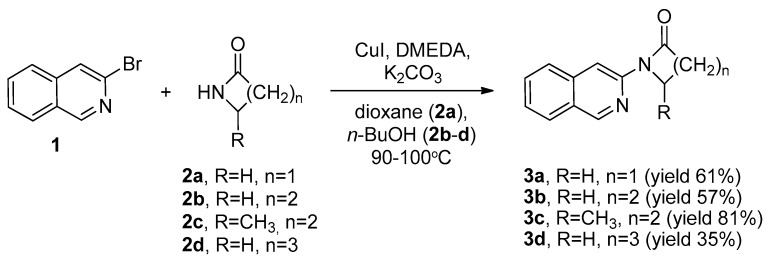
Synthesis of 1-(isoquinolin-3-yl)heteroalkyl-2-ones **3a**–**d**.

Novel isoquinoline derivatives were obtained according to copper-catalyzed Goldberg–Ullmann-type coupling of aryl halides with 2-imidazolidinone [27,28]. The reactions of 3-bromoisoquinoline (**1**) with azetidin-2-one (**2a**), pyrrolidin-2-one (**2b**), 3-methylpyrrolidin-2-one (**2c**), and piperidin-2-one (**2d**) were carried out in the presence of anhydrous potassium carbonate, copper(I) iodide (CuI), and *N*,*N*-dimethylethylenediamine (DMEDA) as a ligand, affording the corresponding 1-(isoquinolin-3-yl)heteroalkyl-2-ones **3a**-**d** (Scheme 1).

**Scheme 2:**
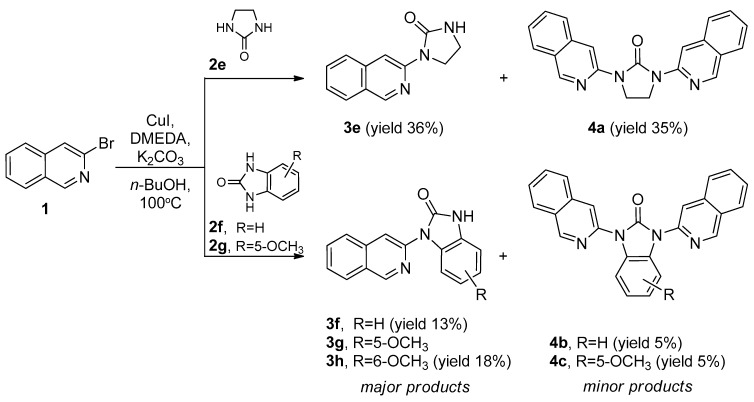
Synthesis of 1-(isoquinolin-3-yl)heteroalkyl(aryl)-2-ones **3e**–**h** and 1,3-di(isoquinolin-3-yl)heteroalkyl(aryl)-2-ones **4a–c**.

In turn, the use of imidazolidin-2-one (**2e**), 1*H*-benzimidazol-2(3*H*)-one (**2f**), or 5-methoxy-1*H*-benzimidazol-2(3*H*)-one (**2g**) as the substrate allowed obtaining the target 1-(isoquinolin-3-yl)heteroalkyl(aryl)-2-ones **3e**–**h** (major products) and the corresponding 1,3-disubstituted derivatives **4a**–**c** (minor products) (Scheme 2).

The formation of the fluorescent product was controlled by sampling the reaction mixture for thin-layer chromatography when the reaction was expected to be completed (3 to 85 h). After the required time, mixtures were cooled to room temperature and diluted with chloroform followed by filtration. Products were separated by preparative thin-layer chromatography (chromatotron). We began by studying the catalytic coupling reaction of 3-bromoisoquinoline with imidazolidin-2-one at different temperatures and various solvents, such as toluene, tetrahydrofuran, dimethylformamide, *n*-butanol, and 1,4-dioxane. It was found that the higher conversion of starting materials and better yields of desired products were achieved using *n*-butanol as a solvent. Reactions using cesium carbonate instead of potassium carbonate as a base were unsuccessful. Synthesis of **3a** required anhydrous 1,4-dioxane at 90 °C, due to limited stability of the substrate **2a**. The use of dimethylformamide as a solvent had a negative effect on the purity and further isolation of the desired products.

In our study, we found that for the 1-(isoquinolin-3-yl)heteroalkyl(aryl)-2-ones a stoichiometric ratio of the reactants in terms of yields and purity was the most important factor. In the synthesis of **3b**, **3d**, **3f**, **3g**, **3h**, and **4a**–**c**, a three-fold excess of compound **2** gave the best result. The equimolar ratio of substrates **1** and **2a** in 1,4-dioxane was used in the synthesis of **3a** giving the product in 61% yield. On the other hand, the highest yield of the product **3e** was achieved in the presence of a five-fold excess of compound **2e** (reaction time 3 h). Importantly, a longer reaction time resulted in an increasing amount of 1,3-di(isoquinolin-3-yl)imidazolidin-2-one (**4a**), even though a large excess of imidazolidin-2-one (**2e**) was used. 

The lowest reactivity was observed in the case of the benzimidazole derivatives **2f** and **2g**, and the yields were diminished (5–18% yield, 80 or 85 h heating at 100 °C). Reaction involving 1-isopropenyl-2-benzimidazolidione under the same conditions (100 °C, 85 h) provided no product. Furthermore, the reaction of **1** with 5-methoxy-1*H*-benzimidazol-2(3*H*)-one (**2g**) afforded a mixture of the two isomeric products: 1-(isoquinolin-3-yl)-5-methoxy-1*H*-benzimidazol-2(3*H*)-one (**3g**) and 1-(isoquinolin-3-yl)-6-methoxy-1*H*-benzimidazol-2(3*H*)-one (**3h**). Due to difficulties associated with purification, compounds **3g** and **3h** could not be isolated in pure form by chromatographic methods. The ^1^H NMR spectrum revealed the 1:1 ratio of the isomers **3g** and **3h**.

### 2.2. Synthesis of 1-(Isoquinolin-3-yl)-3-methylimidazolidin-2-one (***5***) and 1-(Isoquinolin-3-yl)-3-(hetero)aryl-2-ones ***7a***-***d***

**Scheme 3:**
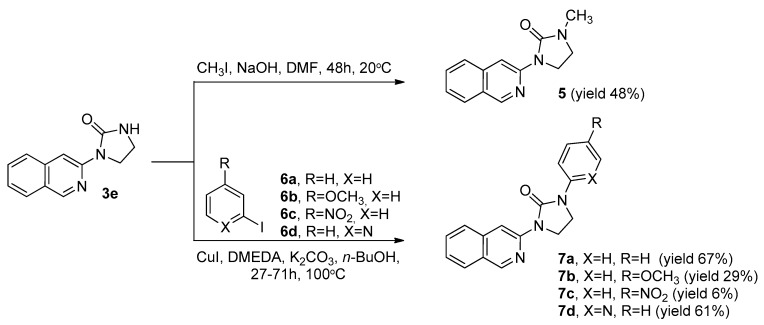
Synthesis of 1-(isoquinolin-3-yl)-3-methylimidazolidin-2-one (**5**) and 1-(isoquinolin-3-yl)-3-(hetero)aryl-2-ones **7a–d**.

The reaction of 1-(isoquinolin-3-yl)-3-imidazolidin-2-one (**3e**) with methyl iodide and solid sodium hydroxide in dimethylformamide at room temperature gave *N*-methyl derivative **5** in moderate yield. Treatment of compound **3e** with aryl or heteroaryl iodides in the presence of copper(I) iodide, *N*,*N*’-dimethylethylenediamine, and potassium carbonate in *n*-butanol at 100 °C gave corresponding 1-(isoquinolin-3-yl)-3-(hetero)aryl-2-ones **7a**–**d** (Scheme 3).

### 2.3. Synthesis of n-Butyl 3-(isoquinolin-3-ylamino)propanoate (***8***) and 3-(Isoquinolin-3-ylamino)-1-(pyrrolidin-1-yl)propan-1-one (***9***)

**Scheme 4:**
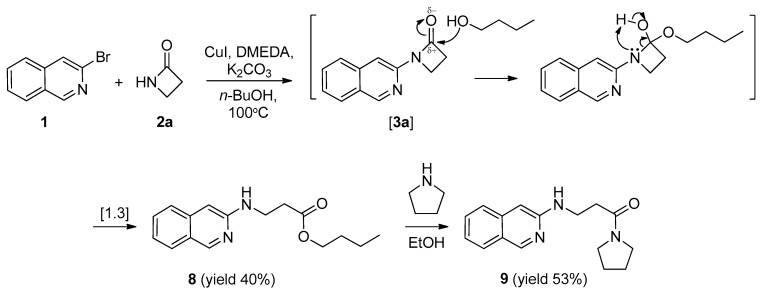
Synthesis of *n*-butyl 3-(isoquinolin-3-ylamino)propanoate (**8**) and 3-(isoquinolin-3-ylamino)-1-(pyrrolidin-1-yl)propan-1-one (**9**).

It was found that the reaction of 3-bromoisoquinoline (**1**) with azetidin-2-one (**2a**) carried out at 100 °C in *n*-butanol led to the formation of the *n*-butyl 3-(isoquinolin-3-ylamino)propanoate (**8**) as shown in Scheme 4. The reaction consists of three mechanistic steps: formation of 1-(isoquinolin-3-yl)azetidin-2-one (**3a**), followed by nucleophilic 1,2-addition of the solvent to the carbonyl group of the β-lactam ring. Finally, *n*-butyl ester **8** is formed as a result of ring-opening of the unstable hemiacetal, followed by [1.3] proton shift. In addition, compound **8** heated under reflux with an excess of pyrrolidine in ethanol gave amide **9** in moderate yield (Scheme 4). It should be noted that in the proposed mechanism, the formation of fluorescent β-lactam **3a** was observed. The compound **3a** was detected by sampling the reaction mixture for thin-layer chromatography and HPLC.

**Figure 1:**
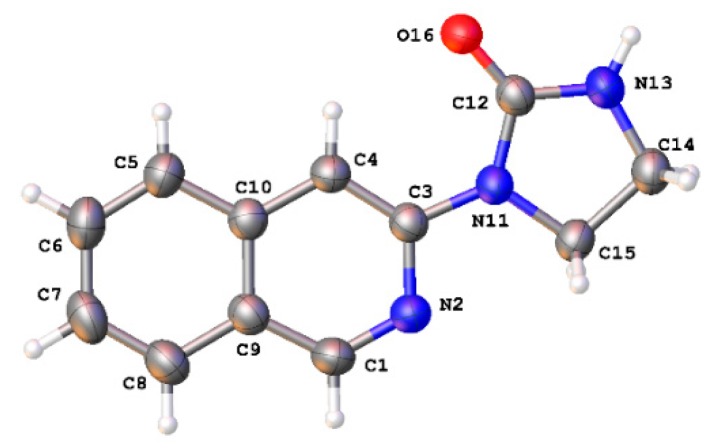
Molecular structure of **3e**. Displacement ellipsoids are shown at the 50% probability level.

**Figure 2:**
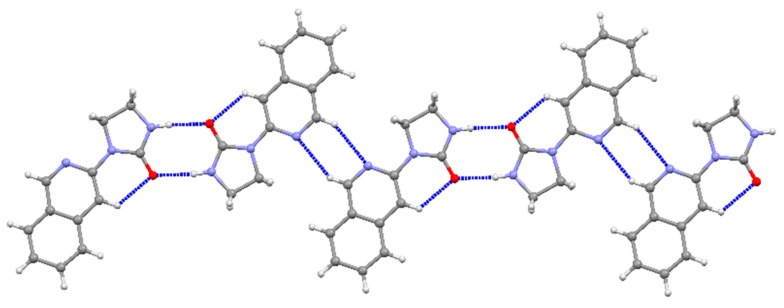
Tapes formed via N-H···O and C-H···N interactions in the crystal structure of **3e**. Short contacts are indicated with dashed lines.

The structures of novel isoquinoline derivatives were confirmed by IR and NMR spectroscopic data, mass spectrometry, and elementary analysis. Moreover, the crystal structure of compound **3e** was determined by X-ray crystallography. The molecule of **3e**, as shown in Figure 1, adopts a strongly flattened conformation with the isoquinoline N atom oriented *trans* relative to the 2-imidazolidinone carbonyl oxygen. The best planes calculated through the isoquinoline and 2-imidazolidinone fragments form a dihedral angle of 3.49°. The *trans* configuration of the 2-pirydyl and 2-imidazolidinone-1-yl fragments about a partially double C-N bond has been found so far in all crystal structures of *N*-(2-pyridyl)imidazolidin-2-one derivatives [29,30,31,32]. It contrasts with the *cis* configuration adopted by this type of molecule in coordination compounds where they act as O,N-chelating ligands [33]. Most probably, the trans configuration of *N*-(2-pyridyl)imidazolidin-2-ones is stabilized by intramolecular C-H···O interaction with a short H4···O16 distance of 2.26 Å. In crystal, the molecules of **3e** are connected through a pair of N-H···O hydrogen bonds (H···O 1.99 Å) between 2-imidazolidinone fragments forming centrosymmetric dimers. These dimers are further connected into tapes by much weaker C-H···N interactions (H···N 2.60 Å) (Figure 2).

Absorption measurements were carried out using a Perkin-Elmer Lambda UV/VIS (Evolution 300 UV-vis) spectrophotometer. Spectra were recorded between 190 and 600 nm at 22 ± 2 °C. The fluorescence spectra of selected compounds **3a**–**f**, **5**, **7a**–**d**, **8**, and **9** were measured under the same conditions. The spectra of the test compounds were recorded in 0.1 M H_2_SO_4_ solution. The photophysical properties data of the selected isoquinoline derivatives (**3a**–**f**, **5**, **7a**–**d**, **8**, and **9**) are summarized in Table 1. Due to the poor solubility in aqueous solution and organic solvents of the di-substituted isoquinolines **4a**–**c** (minor products), their fluorescent properties were not investigated.

The photophysical properties may be strongly affected by solvent properties. In protic hydrogen-bonding solvents such as water, quantum yields are higher compared to hydrocarbon solvents. In case of heteroaromatic compounds, e.g., quinolines or isoquinolines, the hydrogen bond between the lone pair electrons on the nitrogen atom and water stabilizes the ^1^(π,π*) state, destabilizing the ^1^(n,π*) state [34]. This increases the energy gap, reducing the vibronic interaction between the states and results in enhance of fluorescence. The solvent used may have a significant effect on the determined absorption maxima (λ_max_) and the molar absorption coefficient (ε_max_). The most preferred solvent is water, which occurs under physiological conditions and has a permeability limit of 200 nm. In addition, the high polarity of water can cause bathochromic shifts of the bands π → π* and hypsochromic shifts of bands n → π*, associated with groups capable of binding a free electron pair with hydrogen bonds. Thus, compounds **3a**–**e**, **5**, **8**, and **9** were studied in acidic aqueous solution. In the case of compounds **3f** and **7a**–**d,** chloroform was chosen. 

The emission maxima of isoquinoline derivatives **3a**–**f**, **5**, **7a**, **7b**, and **7d** range from 328 to 391 nm (near-ultraviolet region). By contrast, strong electron-withdrawing properties of the NO_2_ group ensures that 1-(isoquinolin-3-yl)-3-(4-nitrophenyl)imidazolidin-2-one (**7c**) has no intrinsic emission (abs_max_ = 350 nm). Fluorescence quenching or decreasing intensity may be observed in the case of nitro compounds [35,36]. 

Compounds **8** and **9** display the same absorption maximum at 406 nm and have favorably long emission wavelengths (494 and 495 nm). A disadvantage of the compounds **8** and **9** is their low quantum yields: 0.223·10^−13^ and 0.053, respectively. Therefore, in the case of **9**, a very weak emission spectrum was observed. 

**Figure 3:**
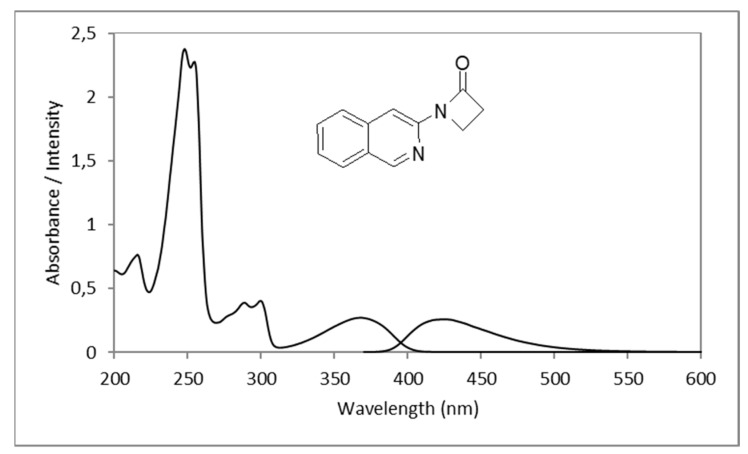
Absorption and fluorescence spectra of 1-(isoquinolin-3-yl)azetidin-2-one (**3a**).

Experiments with isoquinoline derivatives bearing a 4, 5 or 6-membered lactam ring **3a**–**d** provided additional evidence that the absorption and emission spectra are dependent upon their chemical structure. The highest quantum yield value (0.963) was observed for 1-(isoquinolin-3-yl)azetidin-2-one (**3a**), which could be due to the greater structural rigidity. A four-membered ring coupled to the isoquinoline with restricted rotation leads to stabilization of the structure (Figure 3).

Table 1 shows that the fluorescence intensity decreases with increasing heterocyclic ring size. In contrast to the **3a** with the β-lactam ring, compound **3d** with a 6-membered ring displays a lower quantum yield (0.389) and molar extinction coefficient (2541 M^−1^cm^−1^). 

**Figure 4:**
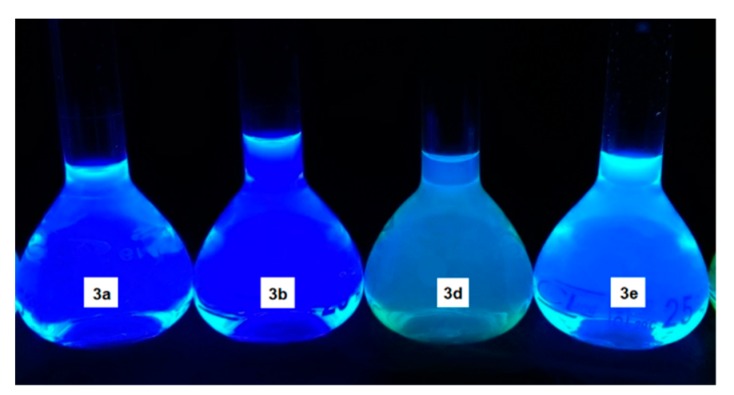
Photograph of fluorescence of compounds **3a**, **3b**, **3d**, and **3e** in 0.1 M H_2_SO_4_ at a concentration of 10^−4^ M under UV (365 nm) light.

**Figure 5:**
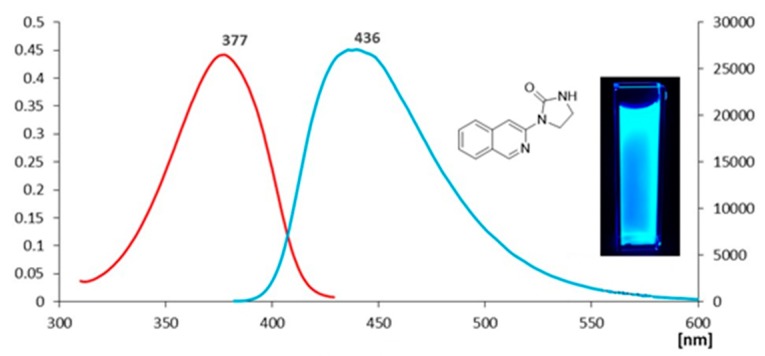
Absorption and fluorescence spectra of 1-(isoquinolin-3-yl)imidazolidin-2-one (**3e**).

Compounds **3a-f** display small Stokes shifts ranging from 54 to 76 nm. Figure 4 shows the fluorescence of compounds **3a**, **3b**, **3d**, and **3e** in 0.1 M H_2_SO_4_. In this group the highest molar extinction coefficient (5083 M^−1^cm^−1^) displays 1-(isoquinolin-3-yl)imidazolidin-2-one (**3e**), whereas the lowest is observed for 1*H*-benzimidazol-2(3*H*)-one derivative **3f** (2032 M^−1^cm^−1^). Compounds **3a-c** and **3e** can be excited at 356, 363, 368, and 377 nm. Figure 5 shows absorption and fluorescence spectra of 1-(isoquinolin-3-yl)imidazolidin-2-one (**3e**). In contrast to compound **3e**, its *N*-methyl analog **5** obtained from **3e** in reaction with methyl iodide has lower fluorescence quantum yield (0.479). The absorption and emission maxima of the *N*-methyl derivative **5** are red-shifted (380 nm and 448 nm).

Due to the lack of solubility of 1-(isoquinolin-3-yl)-3-(hetero)aryl-2-ones **7a**-**d** in water and a 0.1 molar solution of sulfuric acid, the spectra were recorded in chloroform (permeability limit: 250 nm). Compounds **7a**-**d** are characterized by the absorption of electromagnetic radiation in the near-ultraviolet range. The observed absorption maxima are found at the following wavelengths: 302 nm (**7a**), 344 nm (**7b**), 350 nm (**7c**), and 304 nm (**7d**). The heteroaromatic derivative **7d** with the pyridine ring has an electron spectrum very close to its analog **7a** (absorption maxima 304 and 302 nm). The presence of the substituent at the C-4 position of the phenyl ring, both an electron donor (OCH_3_) and an electron acceptor (NO_2_), causes a bathochromic shift of the absorption band (π → π*) by the following values: 42 nm (**7b**) and 48 nm (**7c**). The compounds **7a**, **7b**, and **7d** emit violet light in solution. The attempt to register the emission spectrum of the 4-nitrophenyl derivative **7c** failed. The presence in the structure of the nitro group associated with the aromatic ring causes fluorescence quenching. The determined emission maxima of the compounds **7a**, **7b**, and **7d** are respectively: 391 nm (**7a** and **7b**) and 385 nm (**7d**). In comparison with the unsubstituted compound **7a**, the presence of the methoxy substituent at the C-4 position of the phenyl ring (**7b**) does not change the emission properties. Compound **7a** displays the strongest absorption (λ_max_ = 302 nm) and the molar absorption coefficient (ε_max_) was 34,537 M^−1^cm^−1^. Lower values of the molar absorption coefficient were determined for compounds **7c** and **7d**: 11,072 M^−1^cm^−1^ and 15,581 M^−1^cm^−1^ respectively. The lowest value, ε_max_, of 2251 M^−1^cm^−1^ was characterized by the compound **7b** with a methoxy substituent. Higher values of the Stokes shift were recorded for compounds **7a** (Δυ = 89 nm) and **7d** (Δυ = 81 nm). 

## 3. Experimental Details

**Materials.** Quinine hemisulfate salt monohydrate of ≥98.0% purity suitable for fluorescence was obtained from Sigma-Aldrich. All of the solvents and reagents used in this study were of analytical grade.

**Methods.** Freshly prepared acidic medium 0.1 M H_2_SO_4_ for optical measurements, as well as freshly prepared solutions of compounds, were used. Fluorescence quantum yields were determined in reference to the solution of quinine sulfate in 0.1 M H_2_SO_4_ (Φ_f_ = 0.577, at the wavelength of excitation 350 nm) [37].

The melting points were determined using a Boetius apparatus (VEB Analytik Dresden) and are uncorrected. The infrared spectra were obtained using a Nicolet 380 FTIR spectrophotometer in potassium bromide or in the liquid film. Magnetic resonance spectra (NMR) were recorded with a Varian Gemini 200 BB (^1^H 200 MHz) spectrometer, Varian Unity Inova 500 (^1^H 500 MHz) or Varian Mercury-VX 300 (^1^H 300 MHz) spectrometer. Chemical shifts (*δ*) are reported in ppm relative to residual solvent signals. Coupling constants (*J*) are given in Hz. The mass spectra were recorded on a Shimadzu LCMS 2010 instrument in positive ion mode with an electrospray ionization source (ESI) using a mixture of methanol and acetonitrile containing 0.1% of acetic acid (1:1). The absorption spectra were obtained using a Perkin-Elmer Lambda UV/VIS (Evolution 300 UV-vis) spectrophotometer. The fluorescence spectra were recorded on a Hitachi F-7000 (Scinco FS-2) fluorescence spectrometer.

Compounds were purified by preparative thin-layer chromatography using a Harrison Research Inc. USA chromatotron. Plates were coated with silica gel containing 30% gypsum (Merck Silica Gel PF254). Dichloromethane-ethyl acetate or diethyl ether mixtures as mobile phases were applied. Thin-layer chromatography was performed on silica gel plates with fluorescence detection (Merck Silica Gel 254). After drying, spots were detected under UV light at 254 nm or 365 nm.

*A representative procedure for the reaction of 3-bromoisoquinoline with β, γ, δ-lactams, imidazolidin-2-one, 2-hydroxybenzimidazole or 5-methoxy-1H-benzimidazol-2(3H)-one (***3a–h**, **4a–c***)*

To a stirring solution of 3-bromoisoquinoline (0.104 g, 0.5 mmol) in *n*-butanol (2–3 cm^3^) was added the appropriate lactam (β, γ, δ), imidazolidin-2-one, 2-hydroxybenzimidazole or 5-methoxy-1*H*-benzimidazol-2(3*H*)-one (0.5–2.5 mmol), potassium carbonate (0.207 g, 1.5 mmol), copper(I) iodide (0.01g, 0.05 mmol), and *N*,*N*-dimethylethylenediamine (DMEDA) (0.013 g, 0.15 mmol). For the synthesis of compound **3a**, anhydrous 1,4-dioxane was used at 90 °C. A mixture was heated in an oil bath at 90–100 °C for 3–80 h. The progress of the reaction was controlled by TLC. The mixture was then evaporated under reduced pressure and the residue was extracted four times with 10 cm^3^ portions of chloroform, dried over anhydrous MgSO_4_ and filtered. The solvent was removed in vacuo and the semi-crystalline residue was purified on silica gel by preparative thin-layer chromatography (chromatotron). Thus, the chemical yield of the isolated product was determined.

*1-(Isoquinolin-3-yl)azetidin-2-one (* **3a** *)*

Starting from 0.036 g (0.5 mmol) azetidin-2-one; yield **3a** 0.06 g (61%); reaction time 48 h; eluent: dichloromethane followed by diethyl ether; m.p. 142–146 °C; ν_max_ (KBr, cm^−1^): 3085, 3044, 3003, 2983, 2914, 1739, 1630, 1590, 1466, 1390, 1241, 1138, 849, 752; δ_H_ (200 MHz, CDCl_3_): 3.16 (t, 2H, CH_2_); 3.91 (t, 2H, CH_2_); 7.46 (t, 1H, isoquin.); 7.64 (t, 1H, isoquin.); 7.78 (d, *J* = 8.3 Hz, 1H, isoquin.); 7.89 (d, *J* = 7.9 Hz, 1H, isoquin.); 8.05 (s, 1H, isoquin.); 9.01 (s, 1H, isoquin.); δ_C_ (50 MHz, CDCl_3_): 36.24, 38.30, 107.47, 125.97, 126.52, 126.67, 127.88, 131.16, 137.82, 145.43, 151.72, 165.30; *m/z* (ESI): 199 [M+H]^+^. Anal. Calcd for C_12_H_10_N_2_O (198.22): C, 72.71; H, 5.08; N, 14.13. Found: C, 72.56; H, 5.23; N, 14.01.

*1-(Isoquinolin-3-yl)pyrrolidin-2-one (* **3b** *)*

Starting from 0.128 g (1.5 mmol) pyrrolidin-2-one; yield **3b** 0.06 g (57%); reaction time 65 h; eluent: dichloromethane:ethyl acetate, (19:1 and 9:1, *v*/*v*); m.p. 100–105 °C; ν_max_ (KBr, cm^−1^): 3098, 3063, 3037, 2999, 2967, 2905, 1690, 1624, 1582, 1489, 1448, 1397, 1356, 1292, 1238, 881, 755; δ_H_ (500 MHz, DMSO-*d*_6_): 2.08 (qui, 2H, CH_2_); 2.61 (t, 2H, CH_2_); 4.11 (t, 2H, CH_2_); 7.55 (t, 1H, isoquin.); 7.71 (t, 1H, isoquin.); 7.93 (d, *J* = 8.2 Hz, 1H, isoquin.); 8.06 (d, *J* = 8.2 Hz, 1H, isoquin.); 8.64 (s, 1H, isoquin.); 9.19 (s, 1H, isoquin.); *m/z* (ESI): 213 [M+H]^+^. Anal. Calcd for C_13_H_12_N_2_O (212.25): C, 73.56; H, 5.70; N, 13.20. Found: C, 73.26; H, 5.98; N, 12.86.

*1-(Isoquinolin-3-yl)-3-methylpyrrolidin-2-one (* **3c** *)*

Starting from 0.099 g (1 mmol) 3-methylpyrrolidin-2-one; yield **3c** 0.09 g (80%) as an oil; reaction time 5 h; eluent: dichloromethane:ethyl acetate, (1:1, *v*/*v*); ν_max_ (liquid film, cm^−1^): 3058, 2973, 2926, 1698, 1627, 1584, 1448, 1392, 1236, 1207, 1139, 753; δ_H_ (500 MHz, CDCl_3_): 1.36 (d, *J* = 6.35 Hz, 3H, CH_3_); 1.79–1.85 (m, 1H, CH_2_); 2.37–2.44 (m, 1H, CH_2_); 2.60–2.67 (m, 1H, CH_2_); 2.77–2.84 (m, 1H, CH_2_); 5.04–5.08 (m, 1H, CH); 7.53 (t, 1H, isoquin.); 7.68 (t, 1H, isoquin.); 7.86 (d, *J* = 8.3 Hz, 1H, isoquin.); 7.94 (d, *J* = 8.3 Hz, 1H, isoquin.); 8.55 (s, 1H, isoquin.); 9.12 (s, 1H, isoquin.); δ_C_ (125 MHz, DMSO-d_6_): 20.55, 26.08, 32.35, 54.78, 112.14, 126.50 (two overlapping signals), 127.11, 127.58, 130.88, 137.87, 146.00, 150.70, 174.67; *m/z* (ESI): 227 [M+H]^+^. Anal. Calcd for C_14_H_14_N_2_O (226.27): C, 74.31; H, 6.24; N, 12.38. Found: C, 74.06; H, 6.38; N, 12.20.

*1-(Isoquinolin-3-yl)piperidin-2-one (* **3d** *)*

Starting from 0.149 g (1.5 mmol) piperidin-2-one; yield **3d** 0.04 g (35%); reaction time 5 h; eluent: dichloromethane:ethyl acetate, (9:1, *v*/*v*) followed by diethyl ether; m.p. 81–83 °C; ν_max_ (KBr, cm^−1^): 3060, 2938, 2873, 1655, 1624, 1577, 1481, 1446, 1409, 1352, 1300, 763; δ_H_ (500 MHz, DMSO-d_6_): 1.85–1.93 (m, 4H, 2× CH_2_); 2.48–2.50 (m, 2H, CH_2_), 3.90 (t, 2H, CH_2_); 7.62 (t, 1H, isoquin.); 7.75 (t, 1H, isoquin.); 7.94 (d, *J* = 8.3 Hz, 1H, isoquin.); 8.01 (s, 1H, isoquin.); 8.11 (d, *J* = 8.3 Hz, 1H, isoquin.); 9.24 (s, 1H, isoquin.); *m/z* (ESI): 227 [M+H]^+^. Anal. Calcd for C_14_H_14_N_2_O (226.27): C, 74.31; H, 6.24; N, 12.38. Found: C, 74.48; H, 6.30; N, 12.51.

*1-(Isoquinolin-3-yl)imidazolidin-2-one (* **3e** *)*

Starting from 0.215 g (2.5 mmol) imidazolidin-2-one; yield **3e** 0.038 g (36%); reaction time 3 h; eluent: dichloromethane:ethyl acetate, (8:2, *v*/*v*); m.p. 233–236°C; ν_max_ (KBr, cm^−1^): 3201, 3111, 3053, 2996, 2965, 2902, 1717, 1625, 1583, 1488, 1454, 1415, 1365, 1268, 877, 750; δ_H_ (500 MHz, DMSO-d_6_): 3.43 (t, 2H, CH_2_); 4.11 (t, 2H, CH_2_); 7.20 (s, 1H, NH); 7.44 (t, 1H, isoquin.); 7.64 (t, 1H, isoquin.); 7.83 (d, *J* = 8.2 Hz, 1H, isoquin.); 7.98 (d, *J* = 8.2 Hz, 1H, isoquin.); 8.46 (s, 1H, isoquin.); 9.11 (s, 1H, isoquin.); δ_C_ (125 MHz, DMSO-d_6_): 37.29, 44.56, 105.63, 125.32, 125.59, 126.74, 128.18, 131.33, 137.76, 148.86, 151.31, 159.28; *m/z* (ESI): 214 [M+H]^+^. Anal. Calcd for C_12_H_11_N_3_O (213.24): C, 67.59; H, 5.20; N, 19.71. Found: C, 67.68; H, 5.27; N, 19.56.

Single crystals of **3e** were obtained by crystallization from CHCl_3_. Diffraction data were collected at room temperature with an Oxford Diffraction SuperNova diffractometer using Cu Kα radiation and processed with CrysAlisPro software [38]. Using Olex2 [39], the structure was solved with the program SHELXT [40] and refined by the full-matrix least-squares method on *F*^2^ with SHELXL-2016/6 [41].

Crystal Data for **3e** (C_12_H_11_N_3_O, M = 213.24 g/mol): monoclinic, space group P2_1_/c (no. 14), *a* = 5.9861(1) Å, *b* = 8.4459(1) Å, *c* = 20.2004(2) Å, *β* = 94.6230(10)°, *V* = 1017.97(2) Å^3^, *Z* = 4, *T* = 294 K, μ(CuKα) = 0.750 mm^−1^, *D_calc_* = 1.391 g/cm^3^, 12,358 reflections measured (11.362° ≤ 2Θ ≤ 153.02°), 2115 unique (*R*_int_ = 0.0205, R_sigma_ = 0.0101) which were used in all calculations. The final *R*_1_ was 0.0350 (I > 2σ(I)) and *wR*_2_ was 0.1019 (all data). Illustrations were prepared with the OLEX2 software [39]. CCDC 1,944,714 contains the supplementary crystallographic data for this paper. These data can be obtained free of charge via http://www.ccdc.cam.ac.uk/conts/retrieving.html (or from the CCDC, 12 Union Road, Cambridge CB2 1EZ, UK; Fax: +44 1223 336033; E-mail: deposit@ccdc.cam.ac.uk.

*1-(Isoquinolin-3-yl)-1H-benzimidazol-2(3H)-one (* **3f** *)*

Starting from 0.201 g (1.5 mmol) 1*H*-benzimidazol-2(3*H*)-one; yield **3f** 0.017 g (13%); reaction time 80 h; eluent: dichloromethane:ethyl acetate, (8:2, *v*/*v*); m.p. 186–189 °C; ν_max_ (KBr, cm^−1^): 3183, 3138, 3068, 2902, 2838, 1731, 1628, 1594, 1583, 1486, 1454, 1387, 1177, 873, 741, 720; δ_H_ (500 MHz, DMSO-d_6_): 7.05 (t, 1H, arom.); 7.11 (m, 2H, arom.); 7.69 (t, 1H, isoquin.); 7.75 (d, *J* = 7.7 Hz, 1H, arom.); 7.82 (t, 1H, isoquin.); 8.07 (d, *J* = 8.3 Hz, 1H, isoquin.); 8.20 (d, *J* = 8.2 Hz, 1H, isoquin.); 8.35 (s, 1H, isoquin.); 9.38 (s, 1H, isoquin.); 11.32 (s, 1H, NH); *m/z* (ESI): 362 [M + H]^+^. Anal. Calcd for C_16_H_11_N_3_O (261.28): C, 73.55; H, 4.24; N, 16.08. Found: C, 73.62; H, 4.36; N, 16.18.

*1-(Isoquinolin-3-yl)-5-methoxy-1H-benzimidazol-2(3H)-one (* **3g** *) and 1-(isoquinolin-3-yl)-6-methoxy-1H-benzimidazol-2(3H)-one (* **3h** *)*

Starting from 0.246 g (1.5 mmol) 5-methoxy-1*H*-benzimidazol-2(3*H*)-one; yield mixture of **3g** and **3h** 0.026 g (18%); reaction time 85 h; eluent: dichloromethane:ethyl acetate, (4:1, *v*/*v*); m.p. 164–170 °C; ν_max_ (KBr, cm^−1^): 3165, 3056, 2946, 2901, 2832, 1706, 1628, 1611, 1583, 1495, 1450, 1378, 1269, 1199, 1159, 746; δ_H_ **3g** and **3h** (500 MHz, DMSO-d_6_): 3.72 (s, 3H, OCH_3_); 3.76 (s, 3H, OCH_3_); 6.64–6.67 (m, 2H, 2xarom.); 6.71 (dd, *J*_1_ = 2.2 Hz, *J*_2_ = 8.8 Hz, 1H, arom.); 6.99 (d, *J* = 8.2 Hz, 1H, arom.); 7.38 (d, *J* = 2.2 Hz, 1H, arom.); 7.65–7.71 (m, 2H, 2xisoquin.); 7.77 (d, *J* = 8.8 Hz, 1H, arom.); 7.79–7.84 (m, 2H, 2xisoquin.); 8.04–8.07 (m, 2H, 2xisoquin.); 8.17–8.21 (m, 2H, 2xisoquin.); 8.34 (s, 1H, isoquin.); 8.39 (s, 1H, isoquin.); 9.34 (s, 1H, isoquin.); 9.39 (s, 1H, isoquin.); 11.12 (s, 1H, NH); 11.27 (s, 1H, NH); *m/z* (ESI): 292 [M + H]^+^. Anal. Calcd for C_17_H_13_N_3_O_2_ (291.30): C, 70.09; H, 4.50; N, 14.42. Found: C, 70.31; H, 4.64; N, 14.38.

*1.3-Di(isoquinolin-3-yl)imidazolidin-2-one (* **4a** *)*

Starting from 0.129 g (1.5 mmol) imidazolidin-2-one; yield **4a** 0.03 g (35%); reaction time 19 h; eluent: dichloromethane:ethyl acetate, (19:1, *v*/*v*); m.p. 218–220 °C; ν_max_ (KBr, cm^−1^): 3053, 2926, 2857, 1720, 1628, 1585, 1443, 1393, 1352, 1291, 1244, 872, 737; δ_H_ (500 MHz, DMSO-d_6_): 4.27 (s, 4H, CH_2_-CH_2_); 7.52 (t, 2H, 2xisoquin.); 7.72 (t, 2H, 2× isoquin.); 7.93 (d, *J* = 8.2 Hz, 2H, 2xisoquin.); 8.06 (d, *J* = 8.2 Hz, 2H, 2× isoquin.); 8.64 (s, 2H, 2× isoquin.); 9.21 (s, 2H, 2xisoquin.); *m/z* (ESI): 341 [M + H]^+^. Anal. Calcd for C_21_H_16_N_4_O (340.38): C, 74.10; H, 4.74; N, 16.46. Found: C, 73.89; H, 4.59; N, 16.41.

*1.3-Di(isoquinolin-3-yl)-1H-benzimidazol-2(3H)-one (* **4b** *)*

Starting from 0.201 g (1.5 mmol) 1*H*-benzimidazol-2(3*H*)-one; yield **4b** 0.005 g (5%); reaction time 80 h; eluent: dichloromethane:ethyl acetate, (9:1, *v*/*v*); m.p. 277–278 °C; ν_max_ (KBr, cm^−1^): 3055, 2955, 2925, 1717, 1629, 1594, 1583, 1487, 1450, 1397, 1180, 1156, 740; δ_H_ (200 MHz, DMSO-d_6_): 7.21–7.26 (m, 2H, arom.); 7.69–7.80 (m, 4H, arom.+2× isoquin.); 7.89 (t, 2H, 2× isoquin.); 8.13 (d, *J* = 8.1 Hz, 2H, 2× isoquin.); 8.26 (d, *J* = 7.9 Hz, 2H, 2× isoquin.); 8.43 (s, 2H, 2xisoquin.); 9.47 (s, 2H, 2× isoquin.); *m/z* (ESI): 389 [M + H]^+^. Anal. Calcd for C_25_H_16_N_4_O (388.42): C, 77.30; H, 4.15; N, 14.42. Found: C, 77.21; H, 4.03; N, 14.53.

*1.3-Di(isoquinolin-3-yl)-5-methoxy-1H-benzimidazol-2(3H)-one (* **4c** *)*

Starting from 0.246 g (1.5 mmol) 5-methoxy-1*H*-benzimidazol-2(3*H*)-one; yield **4c** 0.005 g (5%); reaction time 85 h; eluent: dichloromethane:ethyl acetate, (9:1, *v*/*v*); m.p. 189–189 °C; ν_max_ (KBr, cm^−1^): 3050, 2988, 2934, 2898, 2828, 1725, 1629, 1582, 1494, 1447, 1394, 1275, 1179, 737; δ_H_ (500 MHz, DMSO-d_6_): 3.76 (s, 3H, OCH_3_); 6.82 (dd, *J*_1_ = 2.2 Hz, *J*_2_ = 8.8 Hz, 1H, arom.); 7.35 (d, *J* = 2.7 Hz, 1H, arom.); 7.71–7.77 (m, 2H, 2× isoquin.); 7.79 (d, *J* = 8.8 Hz, 1H, arom.); 7.83–7.88 (m, 2H, 2× isoquin.); 8.08–8.12 (m, 2H, 2× isoquin.); 8.23–8.26 (m, 2H, 2× isoquin.); 8.40 (s, 1H, isoquin.); 8.44 (s, 1H, isoquin.); 9.43 (s, 1H, isoquin.); 9.46 (s, 1H, isoquin.); *m/z* (ESI): 419 [M + H]^+^. Anal. Calcd for C_26_H_18_N_4_O_2_ (418.45): C, 74.63; H, 4.34; N, 13.39. Found: C, 74.76; H, 4.48; N, 13.52.

*1-(Isoquinolin-3-yl)-3-methylimidazolidin-2-one (* **5** *)*

To a stirred compound **3e** (0.213 g, 1 mmol) in 1–2 mL of anhydrous DMF was added solid NaOH (0.1 g, 2.5 mmol) and methyl iodide (0.852 g, 6 mmol). After 48 h a mixture was dissolved with chloroform (15 mL) and evaporated to dryness. Compound **5** was separated by use of chromatotron, eluent: dichloromethane:ethyl acetate: methanol, (8:1.5:0.5, *v*/*v*/*v*); yield **5** 0.109 g (48%); m.p. 148–150 °C; ν_max_ (KBr, cm^−1^): 3104, 3037, 2917, 2883, 1702, 1626, 1585, 1504, 1487, 1455, 1432, 1387, 1274, 1228, 878, 763, 748, 733; δ_H_ (400 MHz, DMSO-d_6_): 2.82 (s, 3H, CH_3_), 3.48 (t, 2H, CH_2_), 4.06 (t, 2H, CH_2_), 7.46 (t, 1H, isoquin.), 7.66 (t, 1H, isoquin.), 7.84 (d, *J* = 8.4 Hz, 1H, isoquin.), 8.01 (d, *J* = 8.3 Hz, 1H, isoquin.), 8.48 (s, 1H, isoquin.), 9.13 (s, 1H, isoquin.); δ_C_ (100 MHz, DMSO-d_6_): 31.20, 41.70, 43.90, 105.24, 125.18, 125.37, 126.49, 127.96, 131.11, 137.55, 148.64, 151.12, 157.55; *m/z* (ESI, MeOH:0,1% AcOH+ACN, 1:1, *v*/*v*): 228 [M + H]^+^ and 250 [M + Na]^+^. Anal. Calcd for C_13_H_13_N_3_O (227.26): C, 68.70; H, 5.77; N, 18.49. Found: C, 68.48; H, 6.01; N, 18.61.

*Synthesis of 1-(Isoquinolin-3-yl)-3-(hetero)aryl-imidazolidin-2-ones***7a**-**d**

To a stirred compound **3e** (0.213 g, 1 mmol) in 2–3.5 mL of *n*-butanol was added appropriate aryl iodide (2–5 mmol), copper(I) iodide (0.019 g; 0.1 mmol), *N*,*N*’-dimethylethylenediamine (DMEDA) (0.026 g, 0.3 mmol) and anhydrous potassium carbonate (0.415 g, 3 mmol). A mixture was heated in an oil bath at 90–100 °C for 12–90 h. The progress of reaction was controlled by TLC. Then to a mixture were added 5 mL of water and 5 mL of chloroform and solvents were evaporated under reduced pressure. The residue was extracted five times with 10 cm^3^ portions of chloroform, dried over anhydrous MgSO_4_, and filtered. The solvent was removed in vacuo and semi-crystalline residue was purified on silica gel by preparative thin layer chromatography by use of chromatotron or 20 × 20 cm glass plates (silica gel: 5–17 μm, layer thickness 0.25 mm, medium pore diameter 60Å).

*1-(Isoquinolin-3-yl)-3-phenylimidazolidin-2-one (* **7a** *)*

Starting from 1.02 g (5 mmol; 0.56 mL; 1.823 g/mL) of iodobenzene (**6a**) (added gradually); yield **7a** 0.195 g (67%); reaction time 54 h; eluent: chloroform:ethyl acetate, (4:1, *v*/*v*); m.p. 212–213 °C; ν_max_ (KBr, cm^−1^): 3060, 3025, 2963, 2904, 1704, 1629, 1598, 1586, 1504, 1454, 1410, 1318, 1290, 1250, 735, 685; δ_H_ (300 MHz, CDCl_3_): 3.98 (t, 2H, CH_2_), 4.31 (t, 2H, CH_2_), 7.11 (t, 1H, arom.), 7.36–7.45 (m, 3H, arom.), 7.57–7.67 (m, 3H, arom.), 7.80 (d, *J* = 8.3 Hz, 1H, arom.), 7.87 (d, *J* = 8.2 Hz, 1H, arom.), 8.64 (s, 1H, arom.), 9.02 (s, 1H, arom.); δ_C_ (75 MHz, CDCl_3_): 36.23, 37.32, 102.48, 113.41 (two overlapping signals), 118.41, 120.50, 120.81, 121.93, 122.53, 124.14 (two overlapping signals), 125.66, 133.03, 135.18, 142.78, 145.58, 150.05; *m/z* (ESI): 290 [M + H]^+^ and 312 [M + Na]^+^. Anal. Calcd for C_18_H_15_N_3_O (289.33): C, 74.72; H, 5.23; N, 14.52. Found: C, 74.56; H, 5.01; N, 14.37.

*1-(Isoquinolin-3-yl)-3-(4-methoxyphenyl)imidazolidin-2-one (* **7b** *)*

Starting from 0.702 g (3 mmol) of 1-iodo-4-methoxybenzene (**6b**); yield **7b** 0.092 g (29%); reaction time 27 h; eluent: chloroform:ethyl acetate, (4:1, *v*/*v*); crystallized from methanol; m.p. 166–169 °C; ν_max_ (KBr, cm^−1^): 3003, 2957, 2927, 2847, 1692, 1624, 1581, 1514, 1479, 1461, 1432, 1288, 1247, 1039, 824; δ_H_ (300 MHz, CDCl_3_): 3.81 (s, 3H, OCH_3_), 3.95 (t, 2H, CH_2_), 4.30 (t, 2H, CH_2_), 6.94 (d, *J* = 9.0 Hz, 1H, arom.), 7.41 (t, 1H, arom.), 7.54 (d, *J* = 9.2 Hz, 2H, arom.), 7.59 (t, 1H, arom.), 7.79 (d, *J* = 8.3, 1H, arom.), 7.86 (d, *J* = 8.2, 1H, arom.), 8.63 (s, 1H, arom.), 9.01 (s, 1H, arom.); δ_C_ (75 MHz, CDCl_3_): 41.07, 42.58, 55.51, 107.07, 114.16 (two overlapping signals), 120.19 (two overlapping signals), 125.16, 125.49, 126.67, 127.28, 130.38, 133.19, 137.81, 147.70, 150.30, 155.05, 155.81; *m*/*z* (ESI): 320 [M + H]^+^ and 342 [M + Na]^+^. Anal. Calcd for C_19_H_17_N_3_O_2_ (319.36): C, 71.46; H, 5.37; N, 13.16. Found: C, 71.38; H, 5.34; N, 12.98.

*1-(Isoquinolin-3-yl)-3-(4-nitrophenyl)imidazolidin-2-one (* **7c** *)*

Starting from 0.5 g (2 mmol) of 1-iodo-4-nitrobenzene (**6c**); yield **7c** 0.019 g (6%); reaction time 71 h; eluent: petrol ether:chloroform, (1:1, *v*/*v*) and chloroform:ethyl acetate, (4:1, v/v); m.p. 187–189 °C; ν_max_ (KBr, cm^−1^): 3126, 2955, 2925, 2854, 1710, 1630, 1597, 1507, 1478, 1401, 1327, 1309, 1286, 1246, 1114, 842, 750, 735; δ_H_ (300 MHz, DMSO-d_6_): 4.06–4.11 (m, 2H, CH_2_), 4.24–4.29 (m, 2H, CH_2_), 7.52 (t, 1H, arom.), 7.71 (t, 1H, arom.), 7.88–7.94 (m, 3H, arom.), 8.05 (d, *J* = 8.3 Hz, 1H, arom.), 8.26 (d, *J* = 9.1, 2H, arom.), 8.55 (s, 1H, arom.), 9.19 (s, 1H, arom.); *m*/*z* (ESI): 335 [M+H]^+^ and 357 [M + Na]^+^. Anal. Calcd for C_18_H_14_N_4_O_3_ (334.33): C, 64.66; H, 4.22; N, 16.76. Found: C, 64.58; H, 4.12; N, 16.64.

*1-(Isoquinolin-3-yl)-3-(pyridin-2-yl)imidazolidin-2-one (* **7d** *)*

Starting from 0.41 g (2 mmol; 0.213 mL; 1.928 g/mL) of 2-iodopyridine (**6d**); yield **7d** 0.176 g (61%); reaction time 49 h; eluent: chloroform and chloroform:ethyl acetate, (4:1, *v*/*v*); crystallized from methanol; m.p. 208–209 °C; ν_max_ (KBr, cm^−1^): 3117, 3062, 3000, 2917, 1712, 1627, 1589, 1473, 1435, 1389, 1357, 1287, 1244, 1136, 778, 740; δ_H_ (300 MHz, CDCl_3_): 4.17–4.33 (m, 4H, CH_2_-CH_2_), 6.96–7.00 (m, 1H, arom.), 7.43 (t, 1H, arom.), 7.60 (t, 1H, arom.), 7.69 (t, 1H, arom.), 7.81 (d, *J* = 8.4 Hz, 1H, arom.), 7.87 (d, *J* = 8.2 Hz, 1H, arom.), 8.34 (dd, *J*_1_ = 1.2 Hz, *J*_2_ = 4.4 Hz, 1H, arom.), 8.33–8.39 (m, 2H, arom.), 8.62 (s, 1H, arom.), 9.03 (s, 1H, arom.); δ_C_ (75 MHz, CDCl_3_): 40.77, 41.07, 107.44, 113.30, 118.17, 125.39, 125.69, 126.68, 127.29, 130.42, 137.37, 137.70, 147.30, 147.50, 150.46, 152.22, 154.52; *m*/*z* (ESI): 291 [M+H]^+^ and *m*/*z* = 313 [M + Na]^+^. Anal. Calcd for C_17_H_14_N_4_O (290.32): C, 70.33; H, 4.86; N, 19.30. Found: C, 70.21; H, 4.78; N, 19.22.

*n-Butyl 3-(Isoquinolin-3-ylamino)propanoate (* **8** *)*

To a stirring solution of 3-bromoisoquinoline (0.208 g, 1 mmol) in *n*-butanol (2–3 cm^3^) was added azetidin-2-one (0.213 g, 3 mmol), potassium carbonate (0.415 g, 3 mmol), copper(I) iodide (0.019 g, 0.1 mmol) and *N*,*N*-dimethylethylenediamine (DMEDA) (0.026 g, 0.3 mmol). A mixture was heated in an oil bath at 100 °C for 3 h. The progress of reaction was controlled by TLC. The mixture was then evaporated under reduced pressure and the residue was extracted five times with 10 cm^3^ portions of chloroform, dried over anhydrous MgSO_4_, and filtered. The solvent was removed in vacuo and the residue was dissolved in chloroform (3–5 cm^3^). Compound **8** was isolated on silica gel by preparative thin layer chromatography (chromatotron, eluent: diethyl ether:petrol ether, 6:4, *v*/*v*); green crystals; yield 0.11 g (40%); m.p. 65–67 °C; ν_max_ (KBr, cm^−1^): 3278, 3059, 2959, 2894, 2870, 1736, 1629, 1594, 1543, 1479, 1403, 1363, 1318, 1181, 1105; 810, 749; δ_H_ (500 MHz, DMSO-d_6_): 0.85 (t, 3H, CH_3_); 1.29 (sxt, 2H, CH_2_); 1.52 (qui, 2H, CH_2_); 2.62 (t, 2H, CH_2_); 3.50 (q, 2H, CH_2_); 4.02 (t, 2H, CH_2_); 6.47 (t, 1H, NH); 6.60 (s, 1H, isoquin.); 7.15 (t, 1H, isoquin.); 7.45 (t, 1H, isoquin.); 7.55 (d, *J* = 8.2 Hz, 1H, isoquin.); 7.79 (d, *J* = 8.2 Hz, 1H, isoquin.); 8.85 (s, 1H, isoquin.); δ_C_ (125 MHz, DMSO-d_6_): 14.01, 19.07, 30.62, 34.37, 38.19, 64.06, 96.37, 122.28, 123.01, 124.85, 128.16, 130.61, 138.90, 151.80, 156.05, 172.24; m/z (ESI): 273 [M + H]^+^. Anal. Calcd for C_16_H_20_N_2_O_2_ (272.34): C, 70.56; H, 7.40; N, 10.29. Found: C, 70.42; H, 7.28; N, 10.34.

*3-(Isoquinolin-3-ylamino)-1-(pyrrolidin-1-yl)propan-1-one (* **9** *)*

To a solution of *n*-butyl 3-(isoquinolin-3-ylamino)propanoate (**8**) (0.095 g, 0.35 mmol) in ethanol (3 cm^3^) was added pyrrolidine (0.075 g, 1.05 mmol). A mixture was stirred under reflux (oil bath at 90–100 °C) for 17 h and evaporated in vacuo. Crude product was extracted four times with 10 cm^3^ portions of chloroform, dried over anhydrous MgSO_4_, and filtered. The solvent was removed under reduced pressure and the oily residue was dissolved in chloroform (3 cm^3^). Compound **9** was isolated on silica gel by preparative thin layer chromatography (chromatotron, eluent: ethyl acetate:methanol, 9:1, v/v); green powder; yield 0.05 g (53%); m.p. 141–143 °C; ν_max_ (KBr, cm^−1^): 3280, 3064, 2970, 2872, 1646, 1627, 1593, 1545, 1438, 1220, 814, 752; δ_H_ (500 MHz, DMSO-d_6_): 1.74 (qui, 2H, CH_2_); 1.83 (qui, 2H, CH_2_); 2.56 (t, 2H, CH_2_); 3.28 (t, 2H, CH_2_); 3.37 (t, 2H, CH_2_); 3.46 (q, 2H, CH_2_); 6.35 (t, 1H, NH); 6.59 (s, 1H, isoquin.); 7.14 (t, 1H, isoquin.); 7.45 (t, 1H, isoquin.); 7.54 (d, *J* = 8.3 Hz, 1H, isoquin.); 7.78 (d, *J* = 8.3 Hz, 1H, isoquin.); 8.84 (s, 1H, isoquin.); m/z (ESI): 270 [M + H]^+^. Anal. Calcd for C_16_H_19_N_3_O (269.34): C, 71.35; H, 7.11; N, 15.60. Found: C, 71.51; H, 6.98; N, 15.74.

## 4. Conclusions

In conclusion, we synthesized novel isoquinoline derivatives substituted at position 3 of the heteroaromatic ring in Goldberg–Ullmann-type coupling with appropriate amides in the presence of copper(I) iodide and *N*,*N*-dimethylethylenediamine (DMEDA). Optimization of the coupling reaction of 3-bromoisoquinoline with a series of lactams and imidazolidinones was studied at different temperatures and various solvents. It was found that the higher conversion of starting materials and better yields of desired products were achieved using *n*-butanol. 

The isoquinolines **3a**–**e** and *N*-methyl derivative **5** studied in this report displayed promising fluorescent properties. Compounds **3a**, **3c**, and **3e** possess a higher fluorescence quantum yield (Φ_f_ = from 63.4% to 96.3%) in an acidic aqueous solution than quinine hemisulfate (Φ_f_ = 57.7%) used as a reference standard. Interestingly, compound **3e** with a secondary amide group offering an anchoring group is a prominent scaffold for further functionalization. Moreover, 1-(isoquinolin-3-yl)imidazolidin-2-one (**3e**) displayed the highest molar extinction coefficient (5083 M^−1^cm^−1^). The highest quantum yield value (Φ_f_ = 96.3%) was measured for derivative containing β-lactam ring **3a**. This may be connected with the structural rigidity of this compound. Although these are preliminary results, novel isoquinoline derivatives **3a**–**e** and **5** constitute a promising group of fluorescent compounds with potential applications in medicinal chemistry.

## Figures and Tables

**Table 1:**
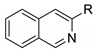
Photophysical properties: molar absorptivity (ε), absorption maxima (λ_max abs._), emission maxima (λ_max em._), Stokes shift (Δ*υ*) and fluorescence quantum yield (Φ_fl_) of the selected isoquinoline derivatives in 0.1 M H_2_SO_4_*.

Comp.	R	ε (M^−1^cm^−1^)	λ_max abs. _(nm)	λ_max em. _(nm)	Δ*υ* (nm)	Φ_fl_
**3a**	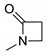	5066	368	425	57	0.963
**3b**	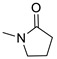	4744	363	417	54	0.559
**3c**	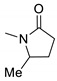	2955	356	417	61	0.954
**3d**	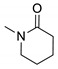	2541	340	416	76	0.389
**3e**	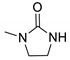	5083	377	436	59	0.639
3f*	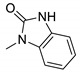	2032	328	383	55	0.101
**5**	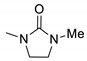	5271	380	448	68	0.479
7a*	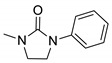	34,537	302	391	89	0.251
7b*	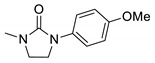	2251	344	391	47	0.443
7c*	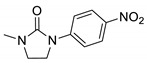	11,072	350	*nd*	*nd*	*nd*
7d*	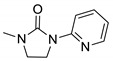	15,581	304	385	81	0.570
**8**	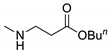	3826	406	495	89	2.23·10^−14^
**9**	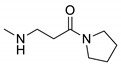	3705	406	494	88	0.053
I-3-A**		*nd*	363	431	68	0.28
QS***		*nd*	(exc. at 350)	*nd*	*nd*	0.577

* compounds were dissolved in chloroform. *nd* - not determined. ** 3-aminoisoquinoline (isoquinolin-3-amine) dissolved in acetonitrile [14]. *** quinine sulfate in 0.1 M H_2_SO_4_ (used as a standard).

## Supplementary Materials

Supplementary data including ^1^H NMR spectra for novel compounds as well as absorption and fluorescence spectra of selected compounds associated with this article are available online. CCDC 1944714 contains the supplementary crystallographic data for this paper. These data can be obtained free of charge via http://www.ccdc.cam.ac.uk/conts/retrieving.html (or from the CCDC, 12 Union Road, Cambridge CB2 1EZ, UK; Fax: +44 1223 336033; E-mail: deposit@ccdc.cam.ac.uk.
