# Supplementary material for: Synthesis and Fluorescent Properties of Novel Isoquinoline Derivatives
Source: Molecules. 2019 Nov 10;24(22):4070. doi: 10.3390/molecules24224070 (PMC6891638; doi:10.3390/molecules24224070)

Supplementary Materials

Synthesis and fluorescent properties of novel isoquinoline derivatives

Łukasz Balewski [1,*] (L.B.), Franciszek Sączewski [1] (F.S., †), Maria Gdaniec [2] (M.G.), Anita Kornicka [1] (A.K.), Karolina Cicha [1] (K.C.), Aleksandra Jalińska [1] (A.J.)

[1] Department of Chemical Technology of Drugs, Faculty of Pharmacy, Medical University of Gdańsk, 80-416 Gdańsk, Poland;
E-Mails: lukasz.balewski@gumed.edu.pl (L.B.); anita.kornicka@gumed.edu.pl (A.K.); cicha.karolina89@gmail.com (K.C.); aleksandra.jalinska@gumed.edu.pl (A.J.)

[2] Faculty of Chemistry, A. Mickiewicz University, 61-614 Poznań, Poland;
E-Mail: magdan@amu.edu.pl (M.G.)

* Author to whom correspondence should be addressed;
E-Mail: lukasz.balewski@gumed.edu.pl; Tel.: +48-58-349-1952; Fax: +48-58-349-1654.

† Deceased 18 October 2018

Copies of IR, NMR spectra of compounds **3b**, **3e**, **4a** *and* **8**

*IR (KBr) spectrum of 1-(isoquinolin-3-yl)pyrrolidin-2-one (**3b**)*

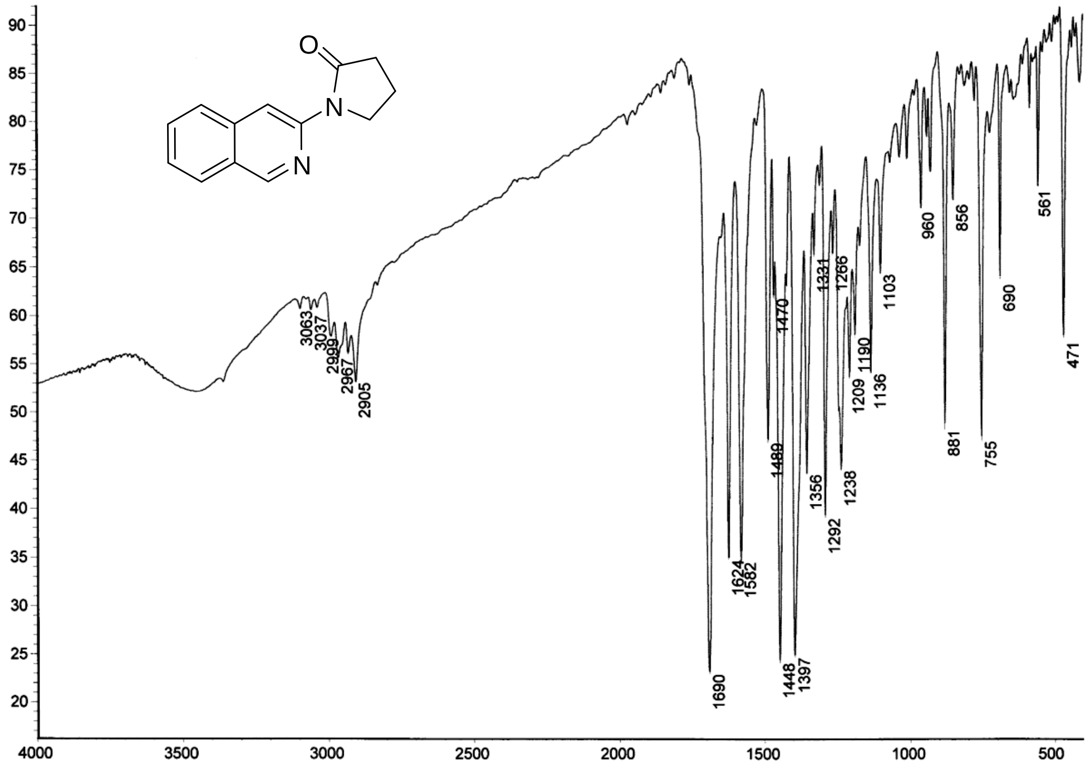

*IR (KBr) spectrum of 1-(isoquinolin-3-yl)imidazolidin-2-one (**3e**)*

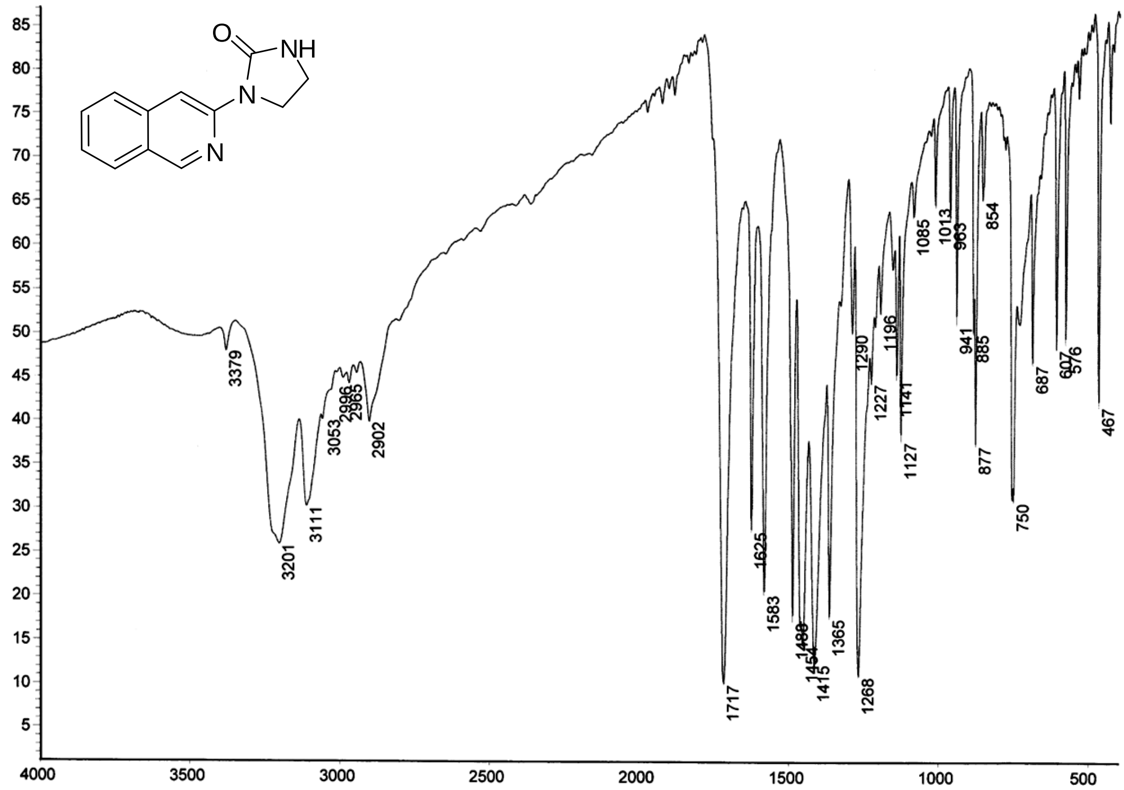

*IR (KBr) spectrum of 1,3-di(isoquinolin-3-yl)imidazolidin-2-one (**4a**)*

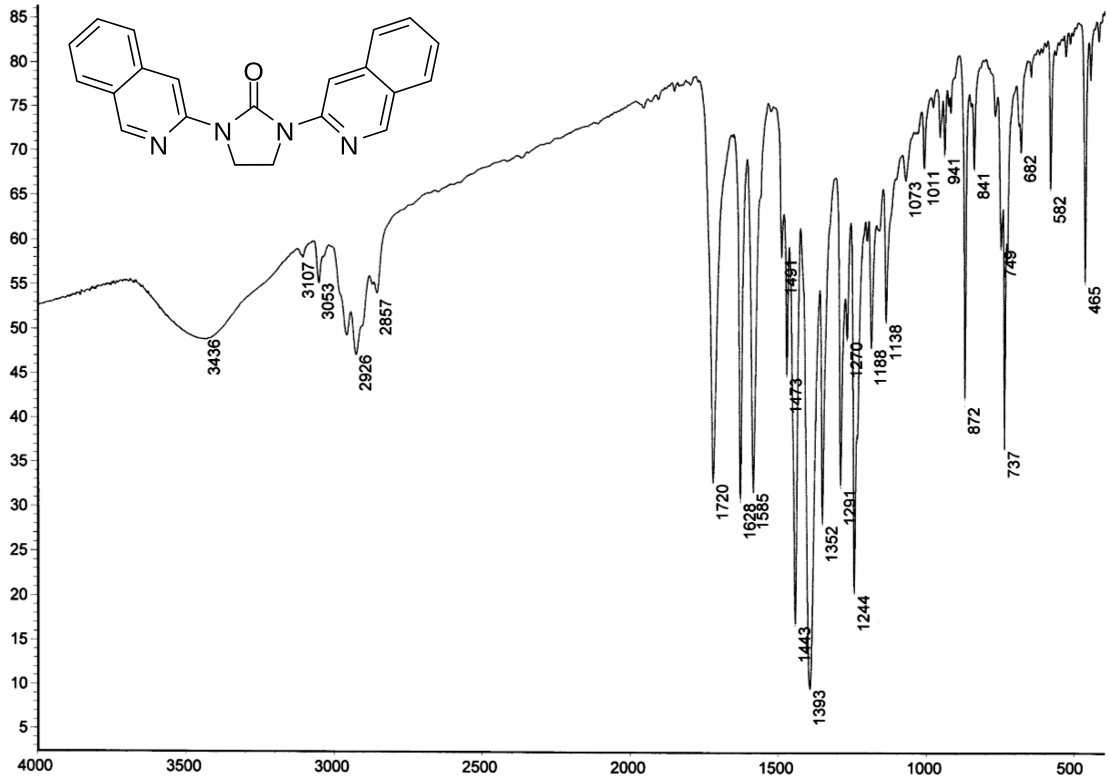

*IR (KBr) spectrum of n-butyl 3-(isoquinolin-3-ylamino)propanoate (**8**)*

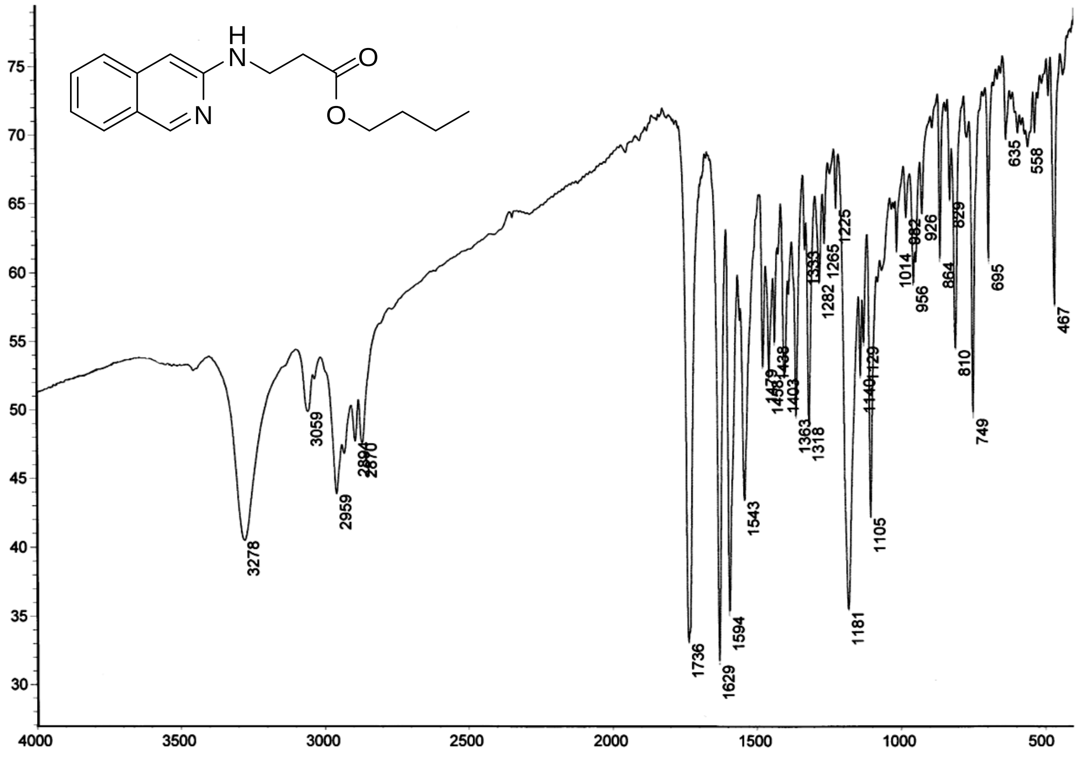

*^{1}H NMR spectrum of 1-(isoquinolin-3-yl)pyrrolidin-2-one (**3b**) in DMSO-d$_6$ (500 MHz)*

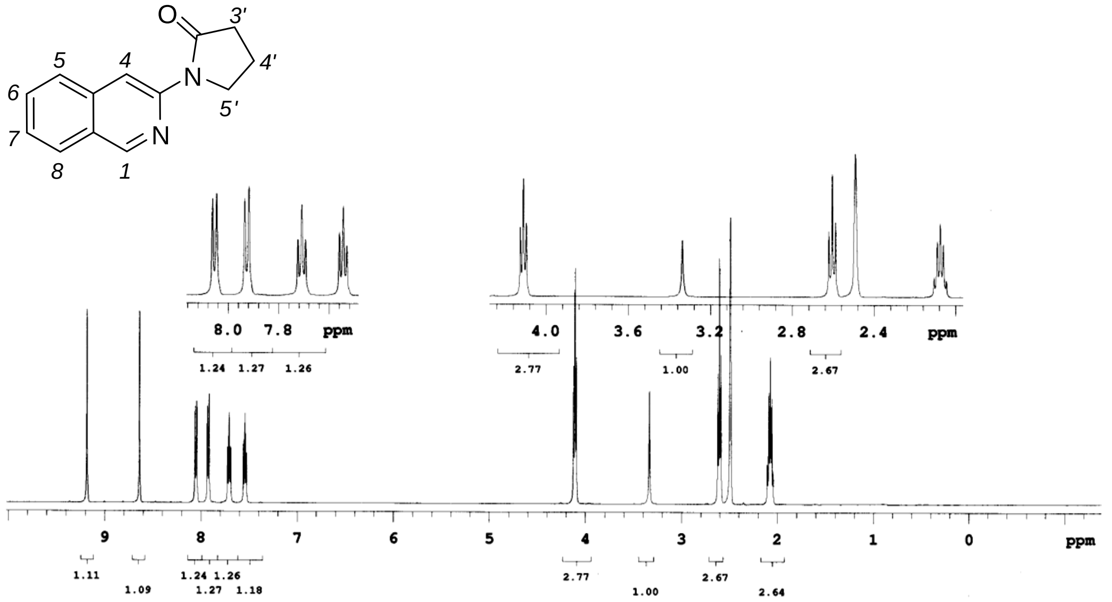

*^{1}H NMR spectrum of 1-(isoquinolin-3-yl)imidazolidin-2-one (**3e**) in DMSO-d$_6$ (500 MHz)*

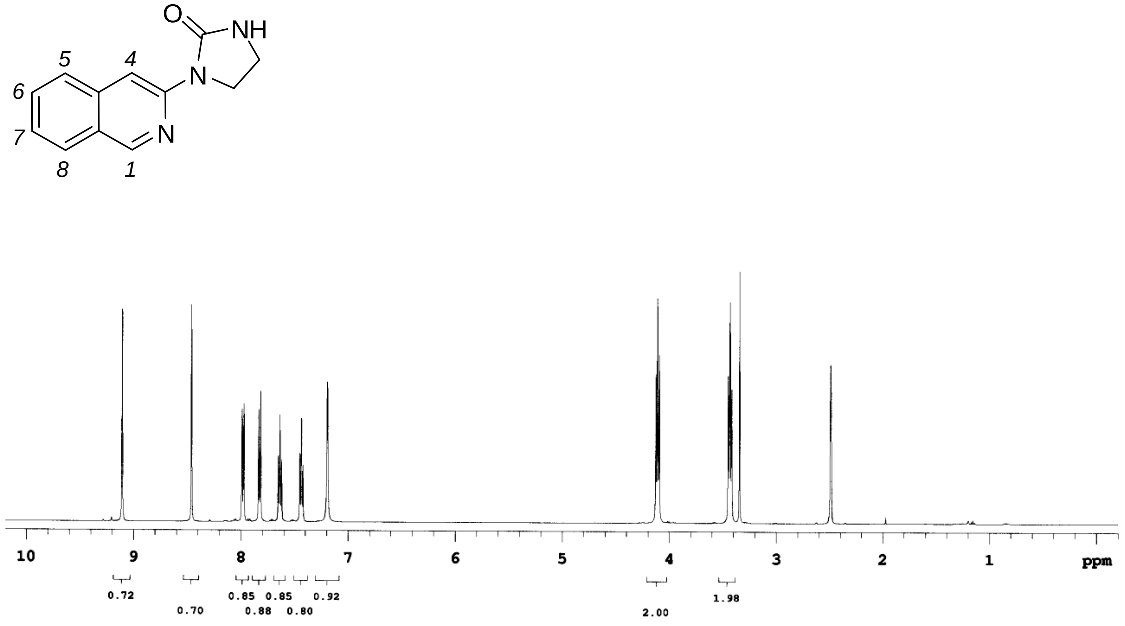

*^{1}H NMR spectrum of 1,3-di(isoquinolin-3-yl)imidazolidin-2-one (**4a**) in DMSO-d_6 (500 MHz)*

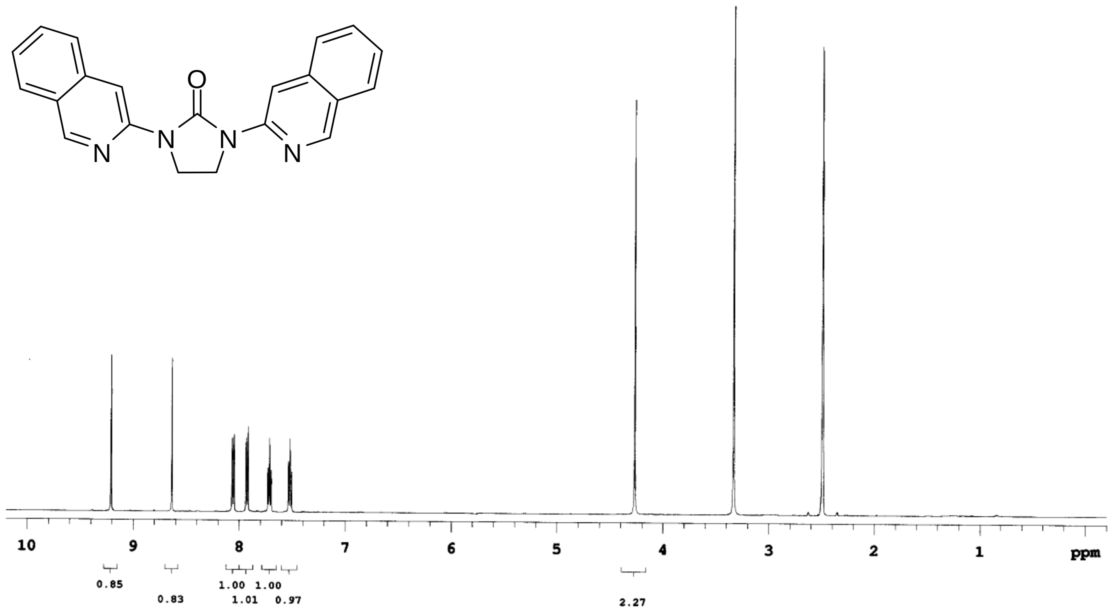

*^{1}H NMR spectrum of n-butyl 3-(isoquinolin-3-ylamino)propanoate (**8**) in DMSO-d_6 (500 MHz)*

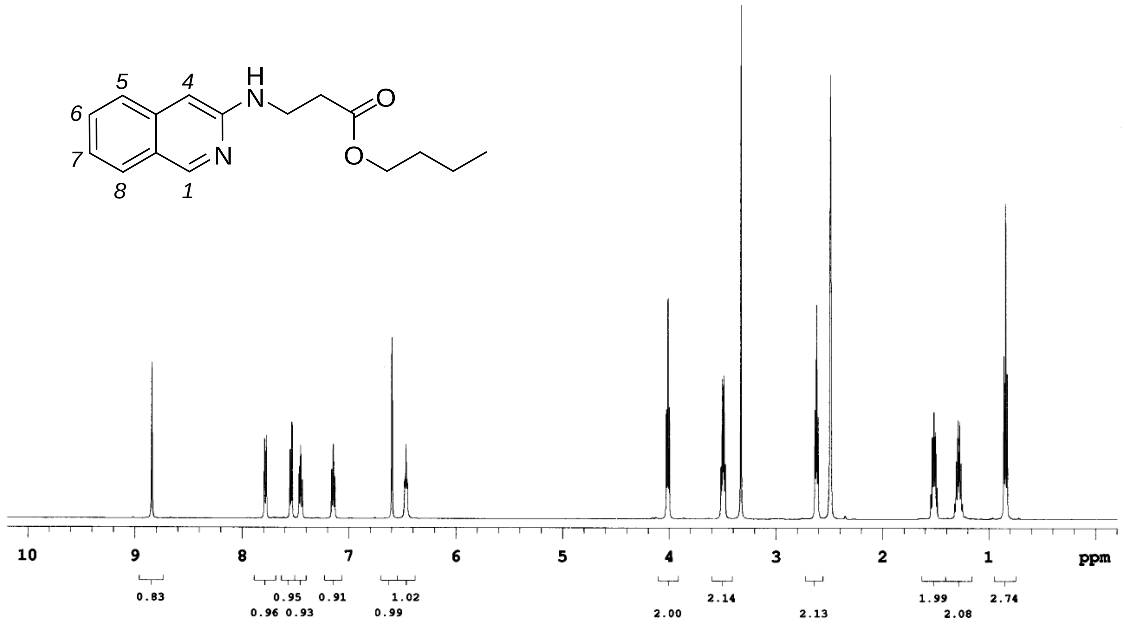

*^{13}C NMR spectrum of n-butyl 3-(isoquinolin-3-ylamino)propanoate (**8**) in DMSO-d_6 (125 MHz)*

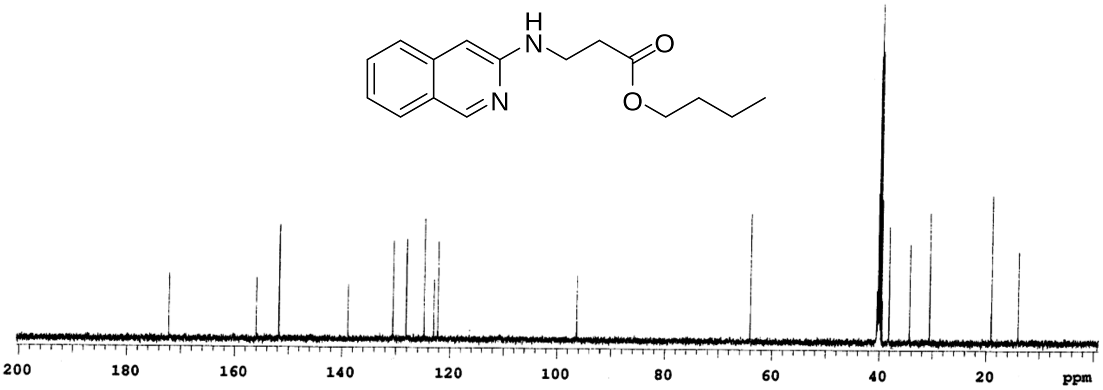

Copies of absorption and emission spectra of compounds **3a**-**f**, **5**, **7a**-**b**, **7d**, **8** and **9**

*1-(isoquinolin-3-yl)azetidin-2-one (**3a**)*

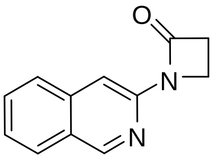

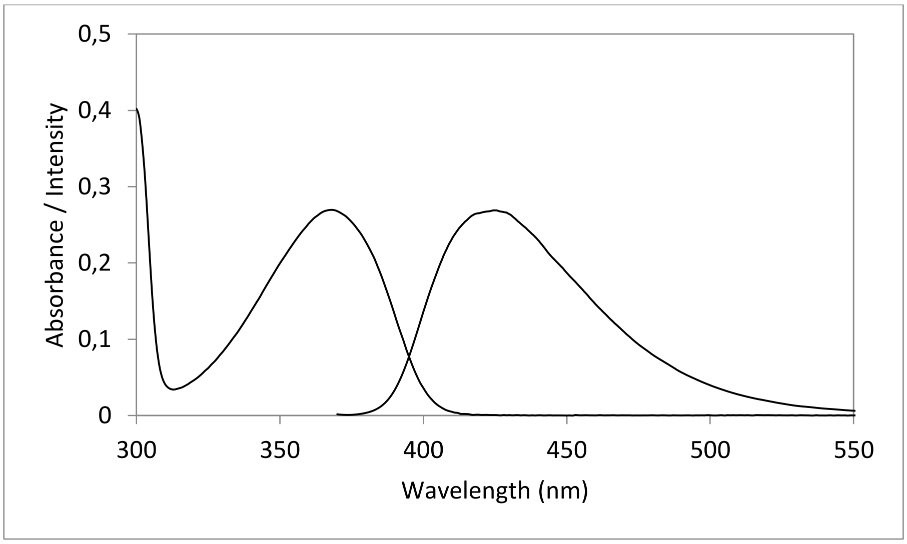

*1-(isoquinolin-3-yl)pyrrolidin-2-one (**3b**)*

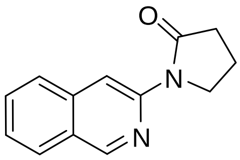

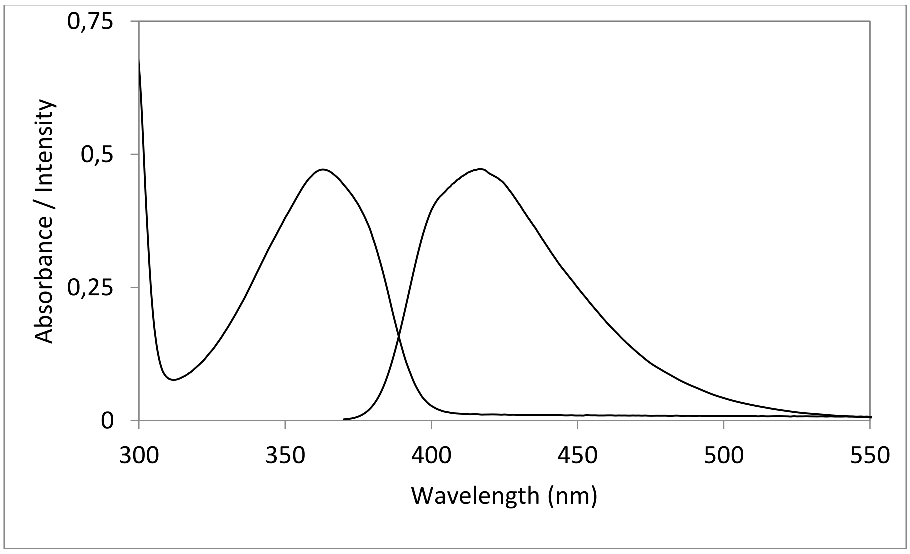

*1-(isoquinolin-3-yl)-3-methylpyrrolidin-2-one (**3c**)*

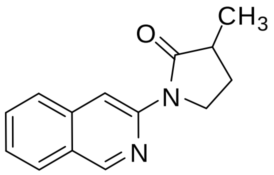

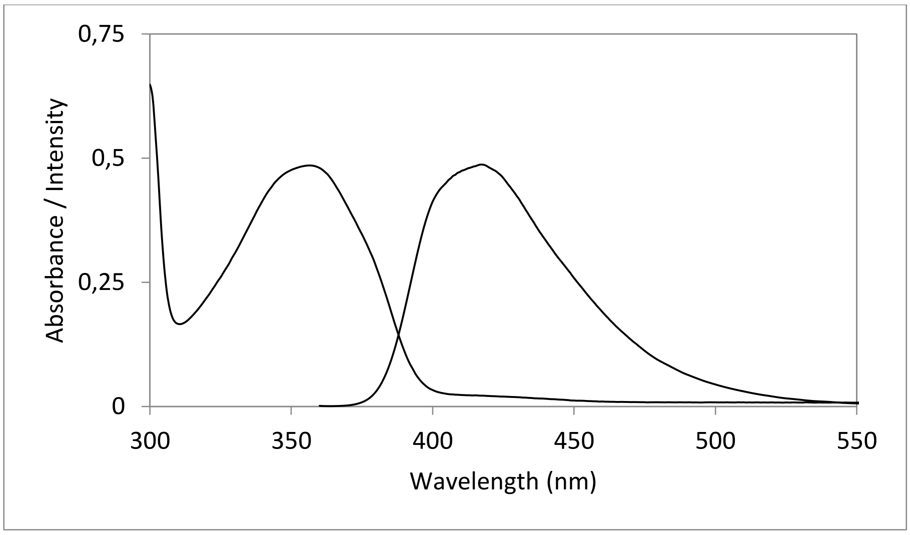

*1-(isoquinolin-3-yl)piperidin-2-one (**3d**)*

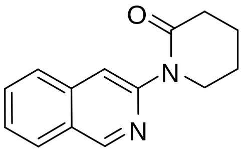

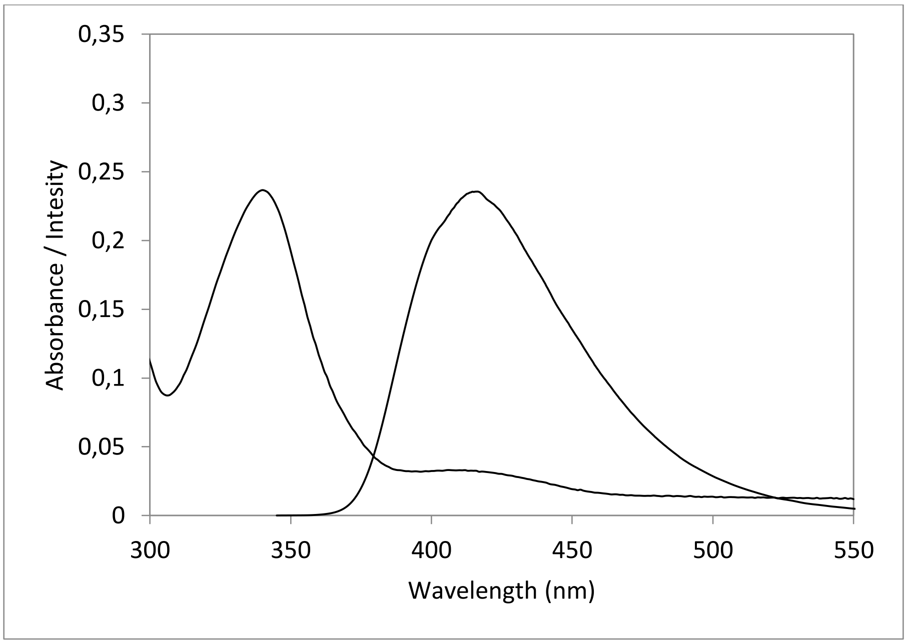

*1-(isoquinolin-3-yl)imidazolidin-2-one (**3e**)*

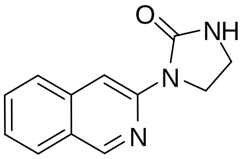

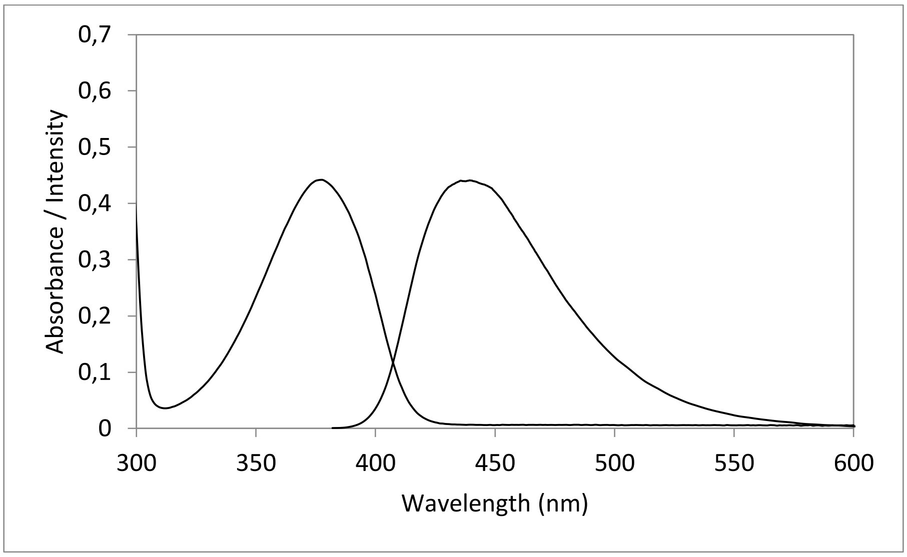

*1-(isoquinolin-3-yl)-1H-benzoimidazol-2(3H)-one (**3f**)*

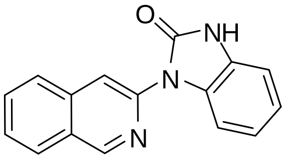

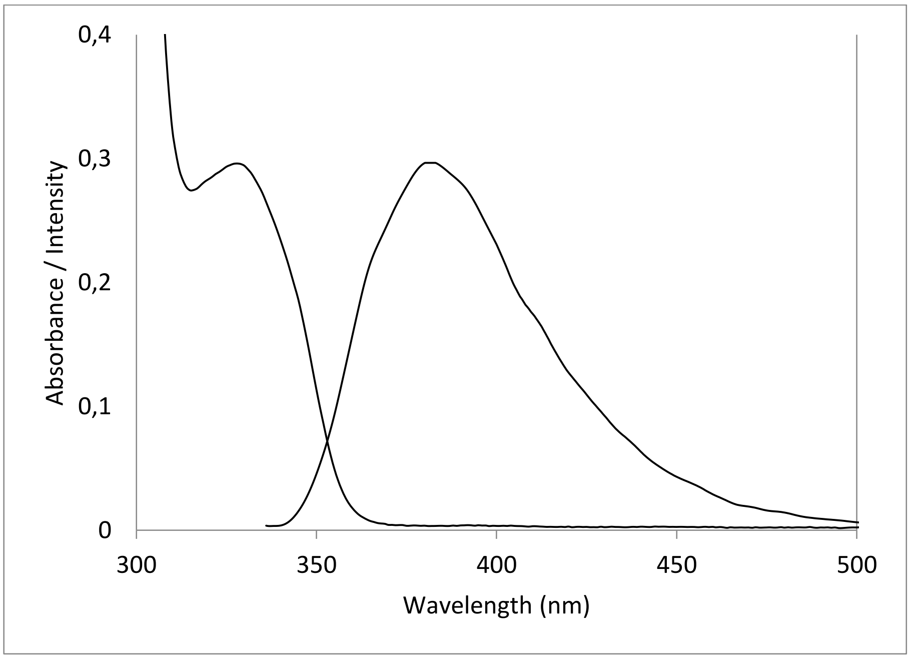

*1-(isoquinolin-3-yl)-3-methylimidazolidin-2-one (**5**)*

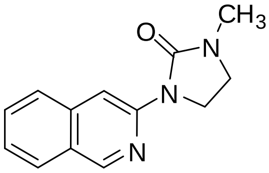

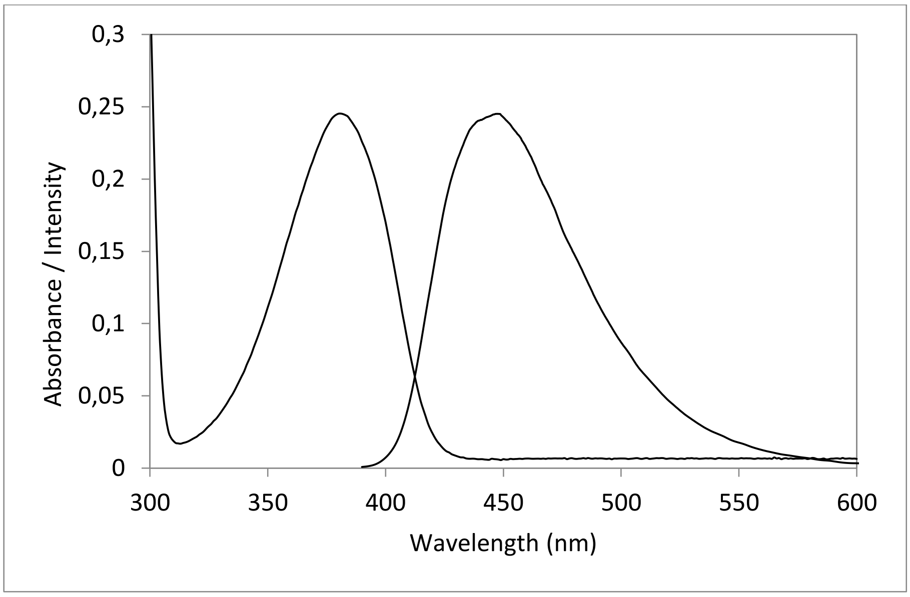

*1-(isoquinolin-3-yl)-3-phenylimidazolidin-2-one (**7a**)*

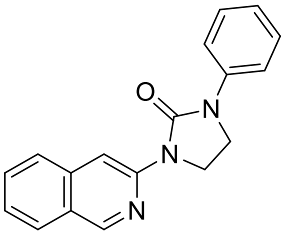

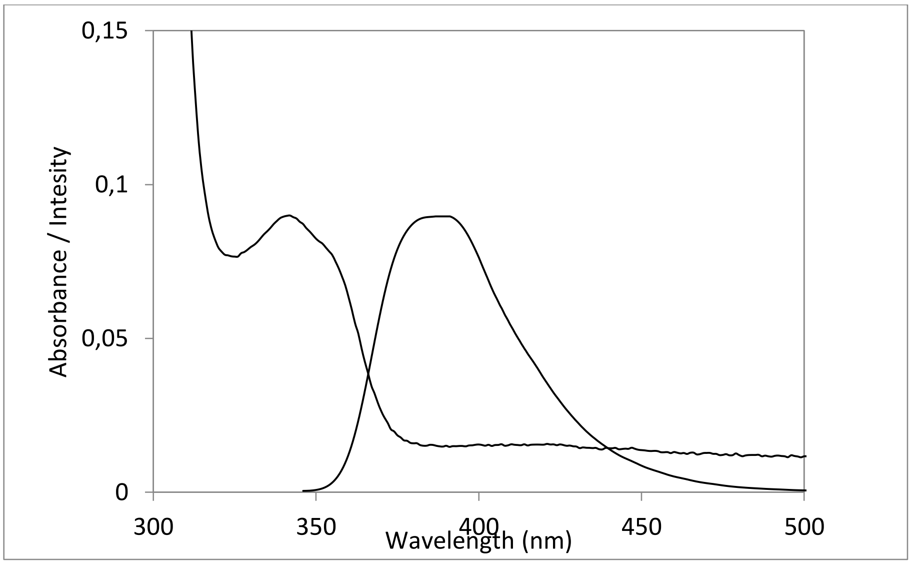

*1-(isoquinolin-3-yl)-3-(4-methoxyphenyl)imidazolidin-2-one (**7b**)*

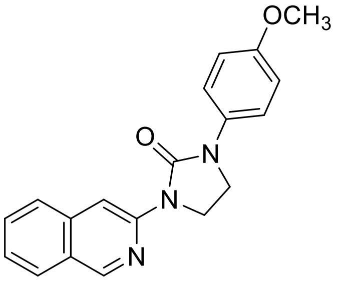

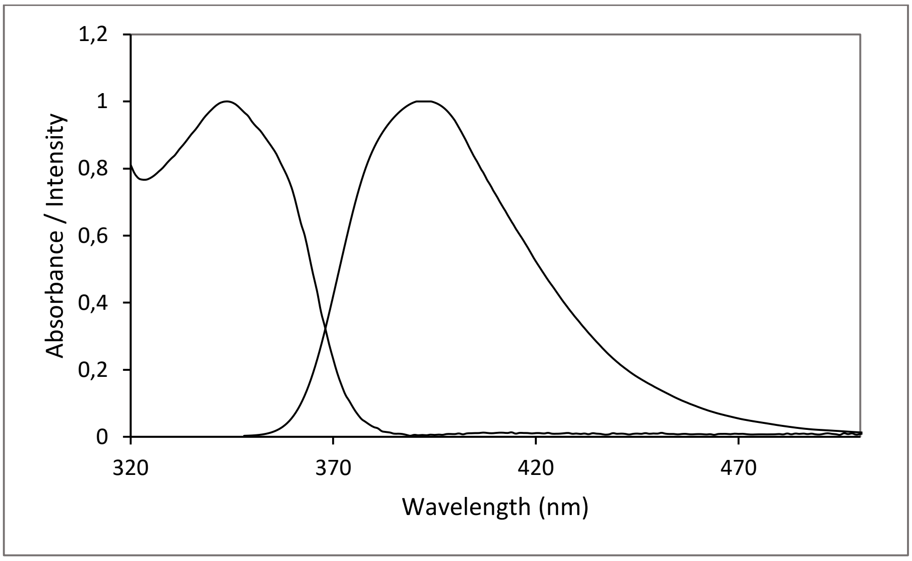

*1-(isoquinolin-3-yl)-3-(pyridin-2-yl)imidazolidin-2-one (**7d**)*

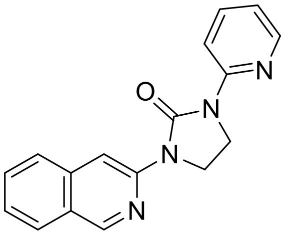

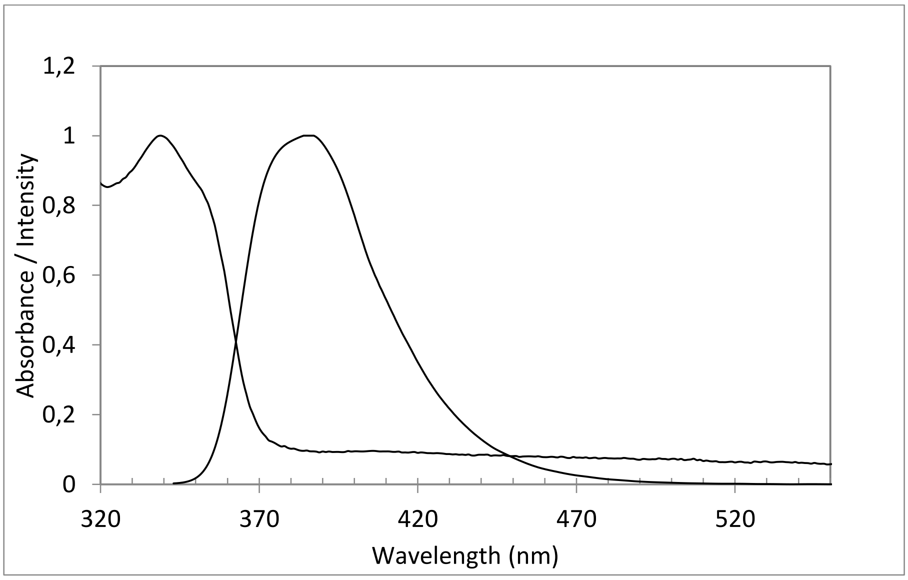

*n-Butyl 3-(isoquinolin-3-ylamino)propanoate (**8**)*

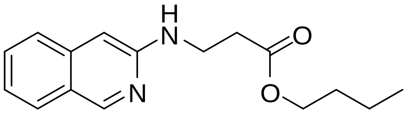

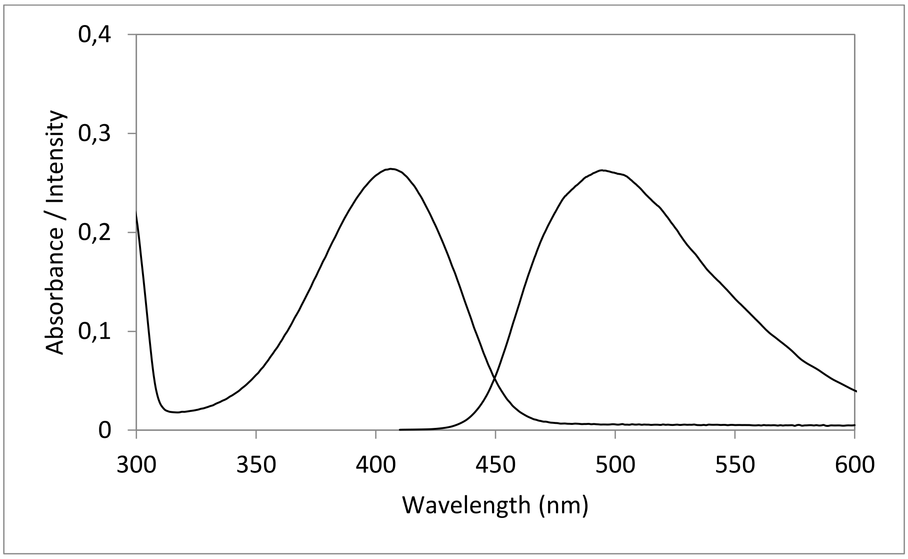

*3-(isoquinolin-3-ylamino)-1-(pyrrolidin-1-yl)propan-1-one (**9**)*

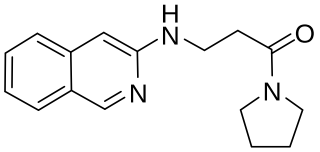

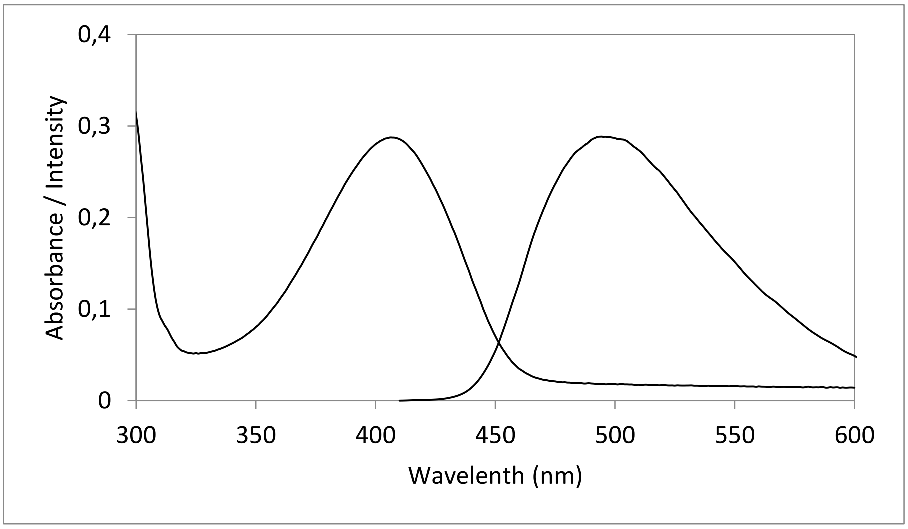

Supplement: Supplementary file 1 [file molecules-24-04070-s001.pdf]
